# A risk-differentiated, community-led intervention to strengthen uptake and engagement with HIV prevention and care cascades among female sex workers in Zimbabwe (AMETHIST): a cluster randomised trial

**DOI:** 10.1016/S2214-109X(24)00235-3

**Published:** 2024-08-14

**Authors:** Frances M Cowan, Fortunate Machingura, M Sanni Ali, Sungai T Chabata, Albert Takaruza, Jeffrey Dirawo, Memory Makamba, Tsitsi Hove, Loveleen Bansi-Matharu, Primrose Matambanadzo, Maryam Shahmanesh, Joanna Busza, Richard Steen, Raymond Yekeye, Amon Mpofu, Owen Mugurungi, Andrew N Phillips, James R Hargreaves

**Affiliations:** a Department of International Health, Liverpool School of Tropical Medicine, Liverpool, UK; b Centre for Sexual Health and HIV AIDS Research (CeSHHAR) Zimbabwe, Harare, Zimbabwe; c Faculty of Public Health and Policy, London School of Hygiene & Tropical Medicine, London, UK; d Institute of Global Health, University College London, London, UK; e Africa Health Research Institute, Durban, South Africa; f National AIDS Council, Harare, Zimbabwe; g Directorate of AIDS and TB, Ministry of Health and Child Care, Harare, Zimbabwe

## Abstract

**Background:**

Female sex workers remain disproportionately affected by HIV. The aim of this study was to determine the effect of risk-differentiated, peer-led support for female sex workers in Zimbabwe on the risk of HIV acquisition and HIV transmission from sex among female sex workers.

**Methods:**

In this cluster randomised, open-label, controlled study, 22 clinics dedicated to female sex workers co-located in government health facilities throughout Zimbabwe were allocated (1:1, through restricted randomisation) to usual care or AMETHIST intervention. Usual care comprised HIV testing, pre-exposure prophylaxis (PrEP), referral to government antiretroviral therapy (ART) services, contraception, condoms, syndromic management of sexually transmitted infections, health education, legal advice, and peer support. AMETHIST added peer-led microplanning tailored to individuals’ risk and participatory self-help groups. All cisgender women (aged >18 years) who had sold sex within the past 30 days and lived or worked within trial cluster areas were eligible. Intervention status was not masked to programme implementers but was masked to survey teams and laboratory staff. After 28 months, a respondent-driven sampling (RDS) survey was done in the female sex worker population around each clinic, which measured the primary outcome, the combined proportion of female sex workers in the surveyed population at risk of transmitting HIV (ie, were HIV positive, not virally suppressed, and not consistently using condoms) or at risk of acquiring HIV (ie, were HIV negative and not consistently using condoms or PrEP). We report prespecified analyses of the disaggregated proportions of female sex workers in the surveyed population at risk of either transmission or acquisition of HIV. Analyses were prespecified, RDS-weighted, and age-adjusted. This trial is registered with the Pan African Clinical Trials Registry, PACTR202007818077777.

**Findings:**

The AMETHIST intervention was started on May 15, 2019, and data were collected from June 1, 2019, until Dec 13, 2021. The RDS survey was done from Oct 18 to Dec 13, 2021, with 2137 women included in the usual care group (11 clusters) and 2131 in the AMETHIST intervention group (11 clusters) after excluding survey seeds (n=132) and women with missing key data (n=44). 1973 (46·2%) of the 4268 female sex workers surveyed were living with HIV; of these, 863 (93·5%; RDS-adjusted) of 931 women in the intervention group and 927 (88·8%) of 1042 in the usual care group were virologically suppressed. 287 (22·4%) of 1200 HIV-negative women in the intervention group and 194 (15·7%) of 1096 in the usual care group reported currently taking PrEP, of whom only two (0·4%) of 569 had protective tenofovir diphosphate concentrations in dried blood spots (>700 fmol/dried blood punch). There was no effect of the intervention on the primary endpoint of risk of both HIV transmission and acquisition (intervention group n=1156/2131, RDS-adjusted proportion 55·3%; usual care group n=1104/2137, RDS-adjusted proportion 52·7%; age-adjusted risk difference –0·9%, 95% CI –5·7% to 3·9%, p=0·70). For the secondary outcomes, the proportion of women living with HIV at risk of transmission was low and significantly reduced in the intervention group (n=63/931, RDS-adjusted proportion 5·8%) compared with the usual care group (103/1041, 10·4%), with an age-adjusted risk difference of –5·5% (95% CI –8·2% to –2·9%, p=0·0003). Risk of acquisition among HIV-negative women was similar in the intervention (n=1093/1200, RDS-adjusted proportion 92·1%) and the usual care group (1001/1096, 92·2%), with an age-adjusted risk difference of –0·6% (95% CI –4·6 to 3·4, p=0·74).

**Interpretation:**

There was no overall benefit of the intervention on combined risk of transmission or acquisition. Viral load suppression in women living with HIV was high and appeared to be further improved by AMETHIST, suggesting potential for impressive uptake and adherence to ART in vulnerable and mobile populations. Sustaining treatment and reinvigorating prevention remain crucial.

**Funding:**

The Wellcome Trust and the Bill & Melinda Gates Foundation.

**Translations:**

For the Shona and Ndebele translations of the abstract see Supplementary Materials section.

**Research in context**

**Evidence before this study**

We searched PubMed with the terms “sex workers”, “ sub-Saharan Africa”, AND (“HIV prevention” OR “HIV prevention cascade” OR “HIV treatment cascade” OR “HIV care cascade”) for articles and conference abstracts published in English from Jan 1, 2000 to March 8, 2024. A 2022 systematic review of female sex workers’ engagement with HIV treatment showed coverage was lower than for adult women in the general population. Research studies of HIV prevention uptake by female sex workers, particularly of new biomedical technologies, are scarce but suggest serious challenges to providing HIV prevention services for sex workers at the scale and intensity necessary for population-level effects. Clinical mentoring of pre-exposure prophylaxis (PrEP) providers has been associated with improved retention in South Africa among female sex workers. One cluster randomised trial in rural Uganda and Kenya tested a dynamic choice model for HIV prevention delivered by community health workers with clinician support; it found biomedical prevention coverage increased by 27·5% among people at risk of HIV, although substantial person-time at risk remained uncovered. Mathematical modelling suggests that the population-attributable fraction of new infections from commercial sex will rise as epidemics contract in the general population. No randomised studies have explored the effects of differentiated support for female sex workers on HIV acquisition and transmission outcomes in Africa or elsewhere.

**Added value of this study**

To our knowledge, this is the first cluster randomised trial to assess effects of risk-differentiated peer support for female sex workers to engage with comprehensive HIV services for the population-level risk of HIV acquisition and transmission and on engagement with HIV prevention and treatment cascades. The effects at the population level were measured through respondent-driven sampling surveys, with self-reported uptake of and adherence to prevention and care confirmed with biomarkers. We found that providing risk-differentiated peer support over and above standard community mobilisation did not affect the overall composite outcome of female sex workers’ population-level risk of HIV acquisition or transmission. Risk of HIV transmission among those living with HIV was significantly and importantly reduced through improved engagement with the HIV care cascade. HIV acquisition risk among HIV negative women, which was very strictly defined, was not affected by our intervention. Effective engagement with the HIV prevention cascade, including in relation to both condoms and oral PrEP, was low.

**Implications of all the available evidence**

Programmes for female sex workers providing risk-differentiated peer support appear to improve care outcomes for sex workers living with HIV, reducing onward transmission. Further enhancing programmes to more effectively enable HIV prevention requires further exploration.

## Introduction

An estimated 15% of new HIV infections in sub-Saharan Africa occur in female sex workers^1^ despite them making up only 1·5% of the general population.^2^ A further quarter occur in their clients and sexual partners.^1^ Despite effective tools for preventing HIV,^3^ poor coverage and suboptimal use of condoms and pre-exposure prophylaxis (PrEP) mean that the risk of acquiring HIV among HIV-negative female sex workers remains unacceptably high. Inadequately supporting female sex workers living with HIV to engage with treatment leads to their high morbidity and mortality, contributing to the risk of transmitting HIV to their sexual partners and children.^2^, ^3^, ^4^ Identifying and implementing strategies to improve access, uptake, and effective use of HIV prevention and treatment among female sex workers remains a global priority.

UNAIDS and WHO recommend targeted sexual and reproductive health services (including HIV services) for sex workers, supported by peer-based community outreach. The optimal delivery model for these services in southern African contexts remains unclear. More generally, community-based models of differentiated service delivery that aim to simplify and decentralise HIV care are being scaled up, having shown effectiveness.^5^ However, how best to differentiate services for different populations and between settings is not well defined. Effectiveness studies of different approaches remain rare.

We developed AMETHIST (Adapted Microplanning: Eliminating Transmissible HIV In Sex Transactions), an intervention package delivered by female sex workers that integrates intensified, systematic community-based support for female sex workers tailored to level of risk, combined with establishing self-help groups (SHGs) that aim to build cohesion between members and with the wider sex-work community, and strengthen psychological and financial resilience. We hypothesised that this theory-based approach would bring about a step change in engagement with both prevention and care compared with the usual standard of care for female sex workers, thereby reducing the proportion of female sex workers at risk of acquiring or transmitting HIV at the population level.

## Methods

### Study design and participants

The AMETHIST trial was a cluster randomised trial nested within Zimbabwe's nationally scaled programme for sex workers, the Key Populations Programme run on behalf of the Ministry of Health and Child Care and National AIDS Council, detailed elsewhere.^6^ 22 clusters were purposively selected from 57 Key Populations Programme sites across Zimbabwe (appendix 3 p 6). Although 23 clusters were eligible, only 22 were required and therefore one cluster was excluded as it was a border town with a transient population. After 28 months, an endline survey was done in all clusters through respondent-driven sampling (RDS). Female sex workers were eligible if they had exchanged sex for money in the past 30 days, were aged 18 years or older, and had been living or working for at least 1 month in that cluster. We nested a mixed methods process evaluation within the trial (appendix 3 p 8).^7^

All cisgender women who sell sex and live or work within trial clusters were eligible to access the Key Populations Programme, either with or without the AMETHIST intervention (intervention group). Written consent was not required for programme participation. Written informed consent, in English, Shona, or Ndebele, was obtained for survey participation before interviews were done and biological samples taken. Funding for the AMETHIST intervention began on April 1, 2018, and for research April 1, 2019 (through different funders). A protocol to randomise clusters to intervention or usual care and assess differences in programme outcomes was approved by the Medical Research Council of Zimbabwe on Oct 1, 2018, before randomisation. Intervention implementation started in May, 2019. With receipt of research funding, a new protocol was developed (without involvement of the funders) including all biobehavioural outcomes described in this Article. This amended protocol was approved by the Liverpool School of Tropical Medicine on Feb 19, 2020 (conditional on Zimbabwean approval). Full approval for the amended trial protocol was received from the Medical Research Council of Zimbabwe on June 10, 2020. No research data were collected before full ethics approval and trial registration.

### Randomisation and masking

Clusters were randomised 1:1 to either the usual Key Populations Programme or the programme with the AMETHIST intervention on Jan 29, 2019, with restricted randomisation (by STC). Restriction factors included province, number of female sex workers seen in the Key Populations Programme in 2017, mean age of first-time attenders, proportion of female sex workers attending the programme who were younger than 20 years, proportion of all attendees aware of HIV status, proportion of all HIV-positive attendees on antiretroviral therapy (ART), and mean number of visits by attendees (appendix 3 p 6). Intervention status was not masked from programme implementers. Survey teams were masked to intervention status and ran surveys in both intervention and control sites. Laboratory staff were unaware of intervention allocation. Data analysis and outcome assessment was not masked.

### Procedures

For the standard care group, the Key Populations Programme provided targeted HIV services (TIDIER framework; appendix 3 p 9), including comprehensive sexual and reproductive health services, HIV testing, and PrEP, following WHO guidelines.^8^, ^9^ Women requiring HIV care were referred to government services. Activities were supported by trained peer educators. Services were provided through the Key Populations Programme in primary care clinics on the same day each week. Outreach workers (salaried social workers) met with peer educators as a group once per month at each site.

For the intervention group, the AMETHIST programme was implemented in addition to usual care. The AMETHIST intervention and its mechanisms of action are shown in the TIDIER framework (appendix 3 p 9) and had two components: peer-led microplanning by female sex workers (consisting of systematic, risk-differentiated, community-based support for all female sex workers in a cluster)^10^, ^11^ and SHGs for 30–40% of female sex workers enrolled in microplanning. Peer microplanners invited female sex workers in their caseload to take part in SHGs until places were filled (and not based on any specific criteria). Peer microplanners managed a geographical hotspot with sex work activity, attempting to recruit all female sex workers working there (50–80 per peer microplanner). They assessed the overall vulnerability of female sex workers (to poor reproductive health outcomes, violence, effects of drinking or substance abuse, etc) in their caseloads every 3 months through a simple risk score and tailored intensity of follow-up accordingly. Female sex workers at high risk were seen once per week, those at moderate risk twice per month, and low risk once per month. Peer microplanners did not ask for information on HIV status; the support they delivered was independent of HIV status. They recorded details of meetings with female sex workers that were used to guide discussions with outreach workers. Outreach workers met with peer microplanners once per week to review their caseload, discuss any challenges, and plan activities for the following week.

SHGs aimed to build social cohesion and psychological and financial resilience among members and the wider female sex worker community. Outreach workers supported peer microplanners to establish and maintain SHGs. The intention was for each microplanner to establish two SHGs for 13–15 female sex workers per group during the trial.

Survey participants were recruited for the endline survey with RDS. In each cluster, we used geographical and social mapping to select six women as so-called seeds: we interviewed these female sex workers and gave each of them two coupons to distribute to peers. Women who received a coupon could attend an interview, and on interview completion were given two coupons for their peers. In all clusters, this process was repeated until at least 200 women were recruited. Participants received US$5 and a further $2 for each woman they recruited. A coupon management system ensured coupons were genuine and minimised repeat participation.

The questionnaire was self-administered, with an audio-computer assisted survey instrument to reduce social desirability bias^12^ and included questions on demographics, sex work, sexual behaviour, condom use, HIV testing history, ART use, stigma, experience of violence, quality of life, mental health, general health, relationships with other sex workers, contact with peer educators or peer microplanners, participation in SHGs, and use of sexual and reproductive health services including through the Key Populations Programme (list of question domains are in appendix 3 p 3). Data were collected onto tablets and uploaded daily. To estimate RDS-2 weights, we asked each participant how many female sex workers aged 18 years and older they knew at the site whom they had seen in the past month and would consider recruiting to the study.

All women had a fingerprick sample collected for HIV and syphilis testing and received results on site. Participants had two dried blood spot samples collected for HIV viral load or PrEP level testing. They were asked to provide two self-administered vaginal swabs for testing of sexually transmitted infections (STIs; *Neisseria gonorrhoea*, *Chlamydia trachomatis*, and *Trichomonas vaginalis*) and, if reporting consistent condom use for the past 2 weeks, for Y chromosomes. Viral load and STI results were made available within 4 weeks of the survey. Free treatment was available.

Fingerprick blood samples for HIV were tested on site according to the Zimbabwe National HIV testing algorithm. The syphilis sample was tested with the DPP Syphilis Screen & Confirm Assay (Chembio Diagnostic Systems, New York, NY, USA). Dried blood spot samples were air dried and stored at room temperature until transported once per week to the Zvitambo Laboratory (Harare, Zimbabwe) for processing and storage.

Women who tested positive for HIV had a dried blood spot sample tested to quantify viral load with the NucliSENS EasyQ HIV-1 version 2.0 (bioMerieux, Lyon, France). HIV-negative women who reported currently taking PrEP had dried blood spot samples sent to the University of Cape Town (Cape Town, South Africa) for tenofovir diphosphate concentrations (protective concentrations defined as tenofovir diphosphate ≥700 fmol/dried blood spot punch and partially protective at 350–700 fmol/punch).

Vaginal samples were kept at 2–8°C and transported to Newlands Clinic laboratory in Harare (Zimbabwe) once per week where they were tested with the Allplex STI Essential Assay Q (Seegene, Seoul, South Korea). Y chromosome samples were tested by the National University of Science and Technology (Bulawayo, Zimbabwe) with the Quantifiler Trio DNA Quantification Kit (Life Technologies, Warrington, UK).

### Outcomes

Our primary composite outcome was the proportion of all survey participants at risk of either HIV acquisition or transmission after 28 months. This outcome was designed to measure the effect of an intervention across the whole population of female sex workers, regardless of HIV status. In our protocol paper^6^ we justified this approach and discussed a number of complexities for interpretation of the proposed composite outcome, including recognition that we might see different effects among HIV-positive and HIV-negative participants. Risk of HIV acquisition is defined as being HIV negative and having any sex in the past month that is not protected by a condom or protective levels of PrEP, taking account of biomarker results. Risk of transmission is defined as being HIV positive with a viral load higher than 1000 copies per mL and having sex in the past month that is not protected by a condom (as previously defined). Appendix 3 (pp 10–11) shows the algorithm for categorising women as HIV negative and at risk of acquisition; HIV negative and not at risk of acquisition; HIV positive and at risk of transmission; or HIV positive and not at risk of transmission.

In addition to the primary outcome, we report a prespecified secondary subgroup analysis, stratifying the main analysis by HIV status prespecified in our analysis plan, reviewed by a data safety monitoring board. Since this analysis involves stratification by HIV status reported in the endline survey, we recognise that these analyses are not randomised comparisons.

We also report engagement with the programme in both intervention and control communities. We report on number of clinic registrations, number of HIV tests done, number of PrEP initiations that took place, number of female sex workers diagnosed with HIV for the first time, and the proportion of these newly diagnosed sex workers who started ART. We were able to compare the proportion of newly diagnosed female sex workers who started ART by group through a simple χ^2^ test.

### Statistical analysis

Our sample-size calculations have been described previously.^6^ We estimated sample size with a k of 0·2 and 0·25. In most scenarios, we estimate 90% power to detect a 30% difference in proportion of female sex workers at risk of HIV acquisition or transmission between study groups. If k is 0·25, we have 78% power to detect a 30% difference from 30% to 21% and over 99% power to detect a 50% difference from 30% to 15%.^6^

Statistical analysis was prespecified and reviewed by the data safety monitoring board (appendix 3 pp 9–10). We assessed evidence of bias in RDS by examining convergence of the primary outcome (appendix 3 pp 11–18). Cluster-level analyses were done accounting for RDS with RDS-2 weighting. We included all RDS participants except seed respondents and those missing key outcome data (ie, HIV test, viral load, tenofovir diphosphate concentration, STI, or Y chromosome data) and did a complete case analysis and weighted the results for each woman in each site by the inverse of her reported network size (ie, the number of other women that she could have recruited).^13^ The target estimand is the difference in transmission or acquisition risk and is derived through an intention-to-treat analysis of a cluster randomised trial.

We described sociodemographic characteristics of the sample at the endline survey and RDS-2-weighted cluster means and ranges by study group. For outcome analyses, we used adapted cluster summaries to estimate risk differences, comparing adjusted and unadjusted means of the RDS-2-weighted site-specific proportions of the binary outcomes in each study group. We adjusted the model for age (prespecified) with the two-step method to adjust for individual-level covariates in the cluster-summary analysis (appendix 3 pp 9–10).^6^ The RDS diagnostics code is in R (version 4.1.3; appendix 3 p 19–20) and was shared with the trial data safety and monitoring board.

We did five sensitivity analyses relating to the primary outcome: first, we dropped one control site that implemented microplanning (ie, the cluster had contamination); second, we ran the analysis without RDS-2 weighting; third, we ran the analysis among those who attended Key Populations clinic (ie, those on treatment); fourth, we used a successive sampling approach to weight the RDS data; and fifth, we used a cutoff of tenofovir diphosphate 350 fmol/punch as protective against HIV acquisition (appendix 3 pp 21–23)

This trial was submitted to the Pan African ClinicalTrials Registry for registration on March 2, 2020. COVID-19 delayed regulation of non-COVID-19 studies. The Pan African Clinical Trials Registry registered the trial on July 2, 2020 (PACTR202007818077777).

### Role of the funding source

The funders of the study had no role in study design, data collection, data analysis, data interpretation, or writing of the report.

## Results

**Figure 1:**
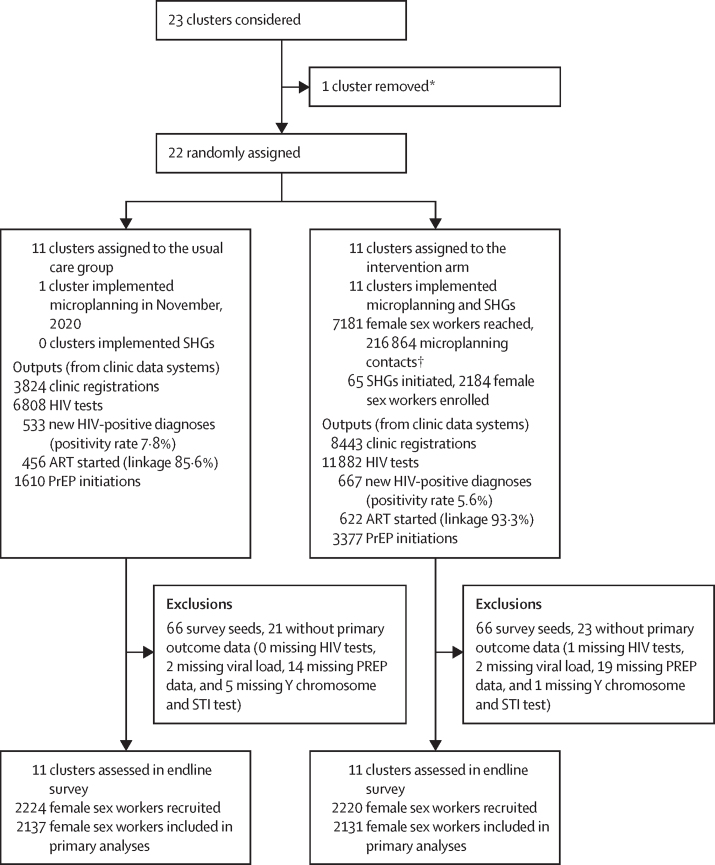
AMETHIST trial profile ART=antiretroviral therapy. PrEP=pre-exposure prophylaxis. SHG=self-help group. STI=sexually transmitted infection. *Only 22 clusters required; one cluster excluded as it was a border town with a very transient population. †Contacts were meetings between a peer microplanner and female sex worker who is listed in her caseload.

The trial ran between June 1, 2019, and Dec 13, 2022. All 22 randomised clusters remained in the study until the end (figure 1). One cluster in the usual care group implemented microplanning from November, 2020, but did not implement SHGs. The RDS endline survey was done from Oct 18 to Dec 13, 2021. Surveys were done in all clusters, with 200–210 female sex workers recruited per cluster. After we excluded women who were seeds (n=132) and women with missing key data (n=44), 2137 women in the usual care group and 2131 in the AMETHIST intervention group were included in the primary outcome analysis.

RDS diagnostics analyses (appendix 3 pp 11–18) found that all seeds started onward recruitment chains, with 116 (88%) of 132 recruitment chains proceeding to five or more waves. There was little evidence of homophily by age in recruitment patterns. Self-reported weighted ego-net analysis suggested that recruitment systematically over-represented both younger and older female sex workers (363 [19%] of 2137 participants in the usual care group and 302 [18%] of 2131 in the intervention group were reported to be age <25 years compared with 429 RDS recruits [22%] of 2137 in the usual care group and 573 [29%] of 2131 in the intervention group; appendix 3 p 20). Homophily might have been more common in intervention sites by primary outcome, although convergence and bottleneck plots of the primary outcome by site suggested waves tended to converge toward the cluster-level summary for most recruitment chains in all clusters (appendix 3 p 20).

The highest proportion of women were aged 20–29 years in both groups, with those in the usual care group older than in the intervention group: median age 32 years (IQR 26–39) compared with 30 years (24–37; table 1). Probably due to their lower age, women in the intervention group were also more likely to be never married, to have sold sex when younger than 18 years, and to have been involved in sex work for less than 1 year. Educational attainment was well balanced between groups. Most participants reported one to five clients per week and having a steady partner (table 1). At endline, HIV prevalence among female sex workers recruited to the RDS surveys was 1041 (48·4%) of 2137 in the control group and 931 (42·7%) of 2131 in the intervention group (table 1). There was no difference in HIV prevalence after adjusting for age (adjusted odds ratio 0·93; 95% CI 0·82–1·06; p=0·31). Microplanning was successfully implemented in all 11 intervention sites between June, 2019, and October, 2021 (table 2), with 7181 female sex workers registered by a peer microplanner and 216 864 interactions with those registered female sex workers recorded (figure 1). Of the sex workers who registered, 6428 (89·5%) had at least one risk assessment and, of these, 2235 (34.8%) were classified by peer microplanners as being at high risk and were seen 2·3 times per month (5023 sessions per 2235 female sex workers; 95% CI 2·1–2·3; target 4 times per month), whereas 1368 (21·3%) women classified as at low risk were seen 0·9 times per month (1273 sessions per 1368 female sex workers; 95% CI 0·92–0·94; target once per month).

**Table 1:** Sociodemographic characteristics of the full RDS at the end of the intervention assessment period

	**Usual care (N=2137)**		**Intervention (N=2131)**		
	Unweighted, n	Mean RDS-weighted, % (range)	Unweighted, n	Mean RDS-weighted, % (range)	Mean percentage point difference
**Age, years**					
18–19	74	4·2% (0·9–8·1)	127	6·8% (1·1–18·2)	2·62%
20–24	355	17·4% (8·2–29·7)	446	21·8% (9·0–34·3)	4·39%
25–29	418	19·8% (13·8–30·3)	441	20·1% (14·0–29·2)	0·33%
30–39	796	36·1% (21·0–48·4)	735	34·1% (18·5–47·1)	−2·08%
≥40	494	22·5% (10·0–43·5)	382	17·3% (5·3–26·0)	−5·26%
**Education level**					
None	76	3·7% (0·5–13·0)	38	1·8% (0·0–4·6)	−1·89%
Primary	478	23·0% (13·7–34·5)	490	23·4% (13·8–37·8)	0·34%
Secondary	1532	71·1% (49·8–81·4)	1539	72·0% (56·8–81·6)	0·91%
≥Tertiary	47	1·9% (0·2–5·1)	63	2·8% (0·4–6·1)	0·82%
Does not know	4	0·2% (0·0–1·8)	1	0·0% (0·0–0·3)	−0·19%
**Marital status**					
Married	113	5·6% (0·5–13·0)	127	6·3% (0·5–11·1)	0·71%
Divorced or separated	1261	58·5% (41·1–70·8)	1147	52·7% (34·2–75·8)	−5·78%
Widowed	325	15·2% (9·0–26·9)	231	10·5% (4·1–15·8)	−4·72%
Never married	438	20·7% (10·8–37·7)	626	30·5% (10·4–49·9)	9·78%
**Age started selling sex, years**					
<18	332	16·0% (7·1–28·9)	418	19·8% (10·9–33·1)	3·87%
18–19	358	17·8% (11·4–33·6)	402	18·5% (7·6–26·4)	0·63%
20–24	631	28·5% (22·7–34·0)	638	29·7% (24·1–33·7)	1·20%
25–29	437	19·6% (9·7–27·5)	404	18·8% (10·2–29·0)	−0·83%
≥30	379	18·1% (6·0–34·9)	269	13·2% (5·4–19·6)	−4·87%
**Duration of sex work, years**					
<1	40	2·1% (0·2–4·9)	48	2·4% (0·3–6·4)	0·29%
1–2	289	14·8% (8·6–25·8)	308	16·1% (7·9–23·1)	1·29%
3–5	490	22·5% (12·5–34·2)	511	24·4% (17·5–29·5)	1·90%
6–9	451	20·8% (16·0–30·4)	451	21·1% (17·1–25·8)	0·25%
10–19	599	27·7% (16·6–37·3)	594	26·3% (17·9–33·3)	−1·33%
≥20	268	12·1% (6·9–18·4)	219	9·7% (2·6–18·2)	−2·39%
**Clients in the previous week, n**					
0	63	2·9% (0·9–5·0)	73	3·8% (0·9–14·7)	0·96%
1–5	1171	57·4% (44·1–71·4)	1216	59·9% (49·1–1·6)	2·44%
6–10	590	26·4% (16·2–39·2)	554	24·0% (16·3–31·1)	−2·37%
11–15	143	5·8% (3·4–7·6)	125	5·4% (1·4–10·7)	−0·43%
16+	170	7·5% (3·1–14·2)	163	6·9% (1·9–12·8)	−0·59%
**Steady partner**					
No	771	34·8% (23·8–56·0)	742	34·7% (23·4–58·1)	−0·16%
Yes	1366	65·2% (44·0–76·2)	1389	65·3% (41·9–76·6)	0·16%
**Attended Key Populations Clinic in the past 12 months**					
No	980	47·4% (30·3–63·2)	833	43·0% (28·5–63·5)	−4·43%
Yes	1157	52·6% (36·8–69·7)	1298	57·0% (36·5–71·5)	4·43%
**Vaccines received for COVID-19**					
None	787	38·4% (7·5–61·1)	759	36·8% (11·5–68·4)	−1·67%
One dose	273	12·2% (8·1–16·5)	230	11·1% (2·3–19·2)	−1·10%
Two doses	1077	49·4% (27·0–83·7)	1142	52·2% (22·9, 82·1)	2·77%
**HIV status**					
Positive	1041	48·4% (34·6–57·4)	931	42·7% (23·8–55·1)	−5·67%
Negative	1096	51·6% (42·6–65·4)	1200	57·3% (44·9–76·2)	5·67%

Participants who were survey seeds are excluded. The mean percentage point differences were calculated by subtracting the mean for the usual care cluster from that for the intervention cluster. The point estimate of each outcome for each cluster is calculated with the RDS-2 methodology. The summary measure provided is a mean of these cluster-level estimates. RDS=respondent-driven sample.

**Table 2:** Fidelity of AMETHIST intervention implementation

	**Reach of delivery**	**Quality of engagement over time**	**Evidence for hypothesised pathways of change**	**Did COVID-19 or other factors disrupt delivery?**
Microplanning	104 empowerment workers trained and deployed; <5 left the programme; 3 of 4 mapping rounds completed across all sites 6 of 8 risk assessments carried out; mean case load was 60 female sex workers (target 50–90)	Differentiated outreach frequency was less than expected; low risk: 0·9 per month (target 1·0); medium risk: 1·4 per month (target 2·0); high risk: 2·3 per month (target 4)	Concerns that risk assessments did not always accurately classify women; peer microplanners and female sex workers expressed fatigue at frequency of expected contacts	Mobility of participants reduced programme retention; violent gang activity in two sites created a climate of fear, reducing engagement; dispersed communities in four sites impeded travel for outreach and clinic visits; power outages reduced telephone communication, reducing contact between peer microplanner and outreach workers during COVID-19 lockdowns; delayed funding interrupted services for up to 6 months COVID-19 led to complete cessation of services for 6 weeks
SHGs	65 of 170 SHGs were established; 30 of 65 SHG still active by the end of programme RDS survey; participants in intervention group were more likely to be SHGs members than those in control group (26·1 *v*s 20·9%; p<0·001)*	Mixed SHG experiences; some groups met regularly and identified group projects, some identified challenges in establishing trust or cohesion	Functioning SHGs coalesced around a shared aim; discussion of health issues was common; some SHGs set up businesses or projects for mutual benefit (eg, communal gardens)	Mobility of participants reduced programme retention; violent gang activity in two sites created a climate of fear, reducing engagement; dispersed communities in four sites impeded travel for outreach and clinic visits; power outages reduced telephone communication, reducing contact between peer microplanner and outreach workers during COVID-19 lockdowns; delayed funding interrupted services for up to 6 months COVID-19 led to complete cessation of services for 6 weeks
Clinic services†	By trial endline, 5673 (79%) of 7181 female sex workers in microplanning groups had registered at a clinic	Frequency of visits was less than intended: clinics received 68% of quarterly visits expected by end of year 2 PrEP stockouts affected uptake (July, 2020 and March, 2021)	Clinic registration, HIV testing, and PrEP initiation were >2 times higher in AMETHIST than control clinics; clinic attendance was higher among SHG members than non-members (1806 [83%] of 2184 members *vs* 3803 [76%] of 4997 non-members; p<0·0001); SHG membership not likely to influence testing between groups (p=0·25)	National lockdown closed all clinics from March 30 to April 21, 2020; travel restrictions affected attendance; health workers experienced burnout; national strike of health-care workers disrupted linkage to ART provision

ART=antiretroviral therapy. PrEP=pre-exposure prophylaxis. RDS=respondent-driven sample. SHG=self-help group.

* There were some community groups not specifically for sex workers and not set up by the Key Populations Programme in the control communities.

† Microplanning and SHGs were intended to increase engagement with clinic services or effect changes in behaviour.

**Figure 2:**
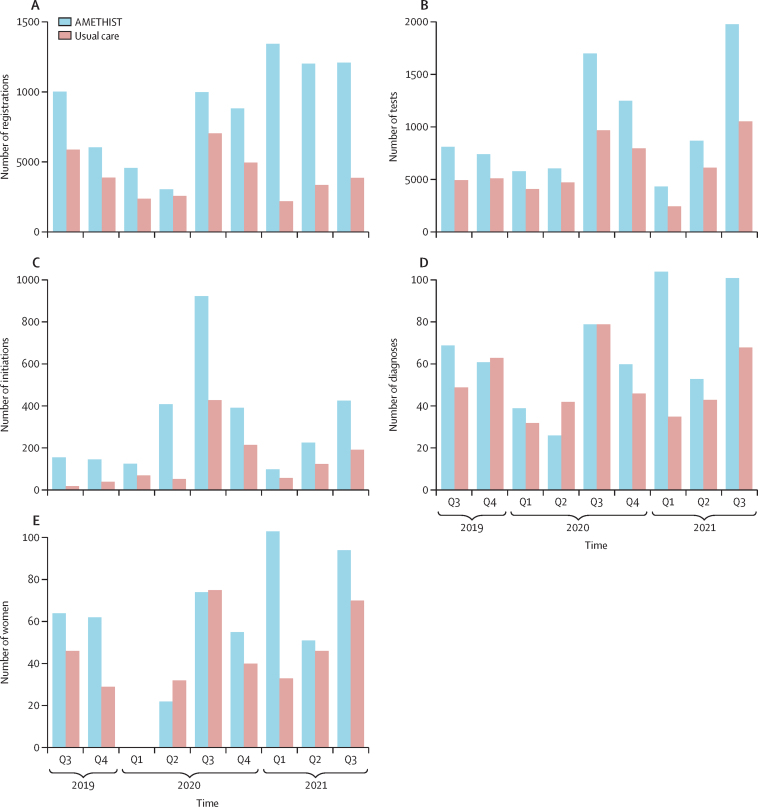
Programme engagement over time in the usual care and intervention groups (A) Clinic registrations over the course of the trial. (B) HIV tests done through the programme clinics over the course of the trial. (C) Pre-exposure prophylaxis initiations in the programme clinics over the course of the trial. (D) New HIV diagnoses made. (E) Newly diagnosed female sex workers starting antiretroviral therapy. Q=quarter.

65 SHGs were established, involving 2184 (30%) of 7181 female sex workers registered with a peer microplanner (figure 1); 30 SHGs remained active at the end of the study (table 2). Microplanning and SHG participation within the intervention group increased engagement with HIV services at Key Populations clinics. SHG members were more likely to have attended a Key Populations clinic in the previous year than non-members (1806 [83%] of 2184 SHG members *vs* 3803 [76%] of 4997 non-members; p<0·0001. Overall, there were more clinic registrations (8443 *vs* 3824), HIV tests (11882 *vs* 6808), HIV diagnoses (667 *vs* 533), ART linkages (622/667 [93·3%] *vs* 456/533 [85·6%]; p<0·0001), and PrEP initiations (3377 *vs* 1610) in the intervention group than control group (figure 2).

The overall crude level of risk of either HIV transmission or acquisition, the primary trial outcome, was 1156 (55·3%) of 2131 women in the intervention group and 1104 (52·7%) of 2137 in the control group (table 3). The age-adjusted risk difference, our primary analysis, was not statistically significant (–0·9%; 95% CI –5·7% to 3·9%; p=0·70; table 3). We calculated the coefficient of variation (k=0·10) and intracluster correlation coefficient (ICC; ICC=0·02) of the primary outcome variable.

**Table 3:** Effect estimates for the primary and secondary outcomes

	**Usual care**		**Intervention**		**Age-adjusted risk difference (95% CI)**	**p value**
	Unadjusted, n	Mean RDS-adjusted, % (range)	Unadjusted, n	Mean RDS-adjusted, % (range)		
**Primary outcome**						
Risk of HIV transmission or acquisition	1104/2137	52·7% (45·7 to 66·0)	1156/2131	55·3% (42·4 to 68·5)	−0·9% (−5·7 to 3·9)	0·70
**Secondary outcomes**						
Acquisition risk among HIV-negative participants	1001/1096	92·2% (84·9 to 96·6)	1093/1200	92·1% (83·6 to 97·3)	−0·6% (−4·6 to 3·4)	0·74
Transmission risk among HIV-positive participants	103/1041	10·4% (4·8 to 15·4)	63/931	5·8% (1·9 to 16·0)	−5·5% (−8·2 to −2·9)	0·0003

Mean respondent-driven sampling proportions are calculated after exclusion of people who were survey seeds and participants with missing primary outcome data (ie, based on the survey at the end of the intervention assessment period, n=4268).

At endline, HIV prevalence among female sex workers recruited to the RDS surveys was 1041 (48·4%) of 2137 in the usual care group and 931 (42·7%) of 2131 in the AMETHIST group (table 1). There was no difference in HIV prevalence after adjusting for age (adjusted odds ratio 0·93; 95% CI 0·82–1·06; p=0·31).

**Figure 3:**
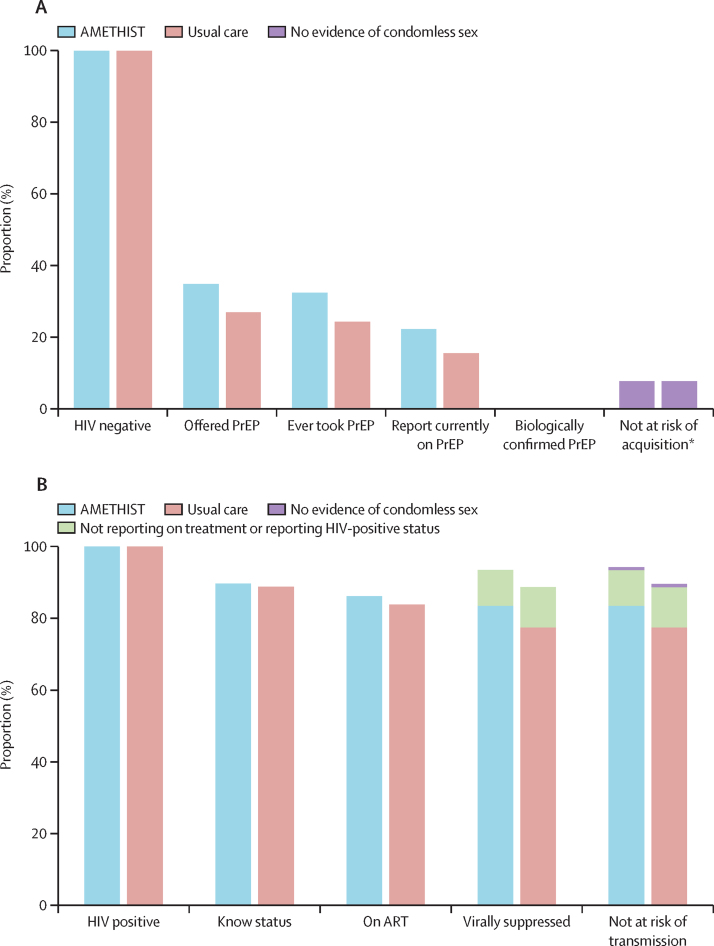
HIV prevention and treatment cascades across usual care and intervention groups The proportion of survey respondents engaging with HIV prevention (A) or treatment (B). ART=antiretroviral therapy. PrEP=pre-exposure prophylaxis. *An additional 93 women who were HIV negative reported currently taking PrEP despite not having reported being offered or ever taking PrEP.

In analysis restricted to women living with HIV, the risk of transmission was significantly lower in the intervention group than in the usual care group (table 3). This relatively low HIV transmission risk reflected that 842 (mean RDS-adjusted proportion 89·7%) of 931 women with HIV in the intervention group and 925 (88·7%) of 1041 in the usual care group knew their status (age-adjusted risk difference 2·1%; 95% CI –2·3 to 6·6). 806 (mean RDS-adjusted proportion 96·2%) of the 842 women who knew they were HIV positive in the intervention group and 877 (94·4%) of the 925 in the control group reported receiving ART (age-adjusted risk difference 2·4%; 95% CI –0·1 to 5·0). Of those receiving ART, 775 (mean RDS-adjusted proportion 96·8%) of 806 in the intervention group and 818 (92·8%) of 877 in the control group were virally suppressed (age-adjusted risk difference 4·3%; 95% CI 2·0 to 6·6). Among all women living with HIV, 863 (mean RDS-adjusted proportion 93·5%) of 931 in the intervention group and 927 (88·8%) of 1042 in the control group were virally suppressed (age-adjusted risk difference 5·8%; 95% CI 2·7 to 8·8; figure 3).

In addition to those virally suppressed, 17 (9·3%) of 183 women across both groups were classified as not being at risk of HIV transmission because they reported consistent condom use across all 13 questions and did not test positive for Y chromosome or *N gonorrhoea* (appendix 3 p 11).

The crude risk of HIV acquisition among HIV-negative women was similar in the intervention and usual care groups (table 3). Among HIV-negative women, 433 (RDS-adjusted proportion 34·9%) of 1200 women in the intervention group and 327 (27·1%) of 1096 in the usual care group reported ever being offered PrEP (age-adjusted risk difference 8·2%; 95% CI –2·9% to 19·3%), 404 (RDS-adjusted proportion 32·4%) of 1200 in the intervention group and 297 (24·3%) of 1096 in the usual care group reported ever taking PrEP (age-adjusted risk difference 8·5%; 95% CI –1·1% to 18·1%), and 287 (RDS-adjusted proportion 22·4%) of 1200 in the intervention group and 194 (15·7%) of 1096 in the usual care group reported currently taking PrEP (figure 2). However, only two (RDS-adjusted proportion 0·4%) of 569 women reporting currently taking PrEP had protective concentrations of tenofovir diphosphate (>700 fmol/punch) in their blood and 39 (RDS-adjusted proportion 5·9%) had concentrations of 350–700 fmol/punch. A further 45 (49·8%) of 94 women were assessed as not being at risk of HIV acquisition because they reported consistent condom use across all 13 questions and did not test positive for Y chromosome or *N gonorrhoea* (appendix 3 p 11). Inconsistent condom use among sex workers in the previous month (regardless of partner type) was high; among 865 (20·3%) of 4268 women reporting no condomless sex in the preceding month (appendix 3 p 12), 574 were tested for Y chromosome (HIV negative or HIV positive with viral load >1000 copies/mL) and 234 (40·8%) tests detected Y chromosome on vaginal PCR.

In further age-adjusted analyses, we found similar effect sizes of the intervention as in the primary analysis strategy when excluding the usual care site with evidence of contamination, restricting to those who reported they had visited a Key Populations clinic (appendix 3 pp 25–27), and when using different approaches to RDS weighting. The effect size was slightly stronger in favour of the intervention when two different cutoff levels were used for biological evidence of PrEP.

## Discussion

In this pragmatic, cluster randomised trial we found no significant effect of the community-supported, risk-differentiated AMETHIST intervention for female sex workers on population-level risk of acquiring or transmitting HIV at 28 months compared with the usual care services available in Zimbabwe. Female sex workers living with HIV in the AMETHIST intervention group were significantly less likely to have transmissible HIV and 93·5% had viral suppression (exceeding UNAIDS 95-95-95 targets) compared with 88·8% in the usual care group. Although risk of acquisition remained high and was similar by group, our definition of acquisition risk was stringent—eg, not allowing for condomless sex with marital partners. Risk-differentiated microplanning was acceptable and feasible to deliver with reasonable fidelity. SHGs were also feasible to implement, although fewer were initiated than planned. Delivery of both components, especially SHGs, was negatively affected by COVID-19 restrictions.

The AMETHIST intervention resulted in improved engagement across the treatment cascade among women living with HIV, resulting in a clinically important and statistically significant reduction in risk of HIV transmission, despite high amounts of cascade engagement among women living with HIV in the usual care group. There was no intervention effect on acquisition risk in HIV-negative women, despite numbers initiating PrEP being twice as high in intervention sites.^14^ Optimal use of HIV prevention was low after accounting for biomarkers and much lower than suggested by programme data or self-reports, with only 0·4% of female sex workers who reported current PrEP use having protective concentrations of tenofovir diphosphate (>700 fmol/punch)^15^ and only 7·9% with concentrations suggestive of current but imperfect PrEP use.^16^

This was a large trial; over 12 000 female sex workers accessed programme services. We used RDS to recruit research participants and recategorised self-reported use of prevention technologies to account for biomarker data. We had a prespecified statistical analysis plan guided by CONSORT principles and integrated a prospective process evaluation to understand strengths and gaps in implementation.

Limitations to our study included that intervention and research funding were provided separately and at different start times. We were thus unable to do a baseline survey and instead used routine service data from trial clinics to inform restricted randomisation. After randomisation, service data suggested that sites in the AMETHIST intervention group had more female sex workers and a higher proportion of younger female sex workers than usual care sites (appendix 3 p 7). We carried out a number of preplanned sensitivity analyses (appendix 3 pp 25–27). Our findings were robust to these additional analyses. In none of the sensitivity analyses was there an effect of the AMETHIST intervention on HIV acquisition, only on transmission.

There was no evidence of an effect of the intervention influencing whether a person has HIV at endline. HIV prevalence was similar between groups after age adjustment. Although our primary outcome is defined on the basis of HIV status, which was ascertained after randomisation, our aim was to test the effect of a status-neutral intervention in the whole population. Our primary outcome is not stratified by HIV status, so its validity is not affected by any possible effect of the intervention on incidence. Nevertheless, we recognise that our separate analyses by HIV status are not randomised. However, we consider any resulting bias likely to be small. For women who were HIV positive, most would have been infected before the intervention started, so any influence of the intervention would be modest. For women who were HIV negative at endline, the characteristics of those who are HIV negative would differ slightly between groups if the intervention reduces incidence, but we similarly cannot envisage more than a minor effect.

Another limitation is that the trial took place during the COVID-19 pandemic. Zimbabwe had long-term restrictions. Although the Key Populations Programme was a permitted essential service, restrictions on movement reduced clinic opening hours and the number of women able to access services per day. The programme adapted by transferring services from clinic to the community where possible, but effects on service provision were inevitable. The COVID-19 pandemic also negatively affected implementation of SHGs, with fewer established than intended and challenges to sustaining them. These issues with the SHGs probably weakened the intervention's effect on social cohesion and building support networks. Our process evaluation suggests that when SHGs continued to operate, they appeared to enhance engagement with clinical services and improved motivation and self-efficacy of members to do so.

We used RDS, which has known limitations, to recruit female sex workers.^17^, ^18^ Reviewing our RDS diagnostic data suggested no systematic differences in recruitment by group although homophily might have been more common in intervention group sites by primary outcome (appendix 3 p 20). Our comparison of ego-nets within the whole sample suggests that our RDS systematically under-recruited both younger and older female sex workers and over-recruited women who have heard of the Key Populations Programme, but this finding does not differ by group. This sampling issue is similar to findings in our previous SAPPH-IRe trial;^19^ although it highlights a need for caution, it is not suggestive of bias in cross-group comparison.

Although the absence of effect on our primary endpoint is disappointing, this was a composite endpoint that combined both risk of HIV acquisition and HIV transmission. The improvements in treatment coverage are important and support other evidence for how proactive identification of hidden populations living with HIV and linkage to care are successful.^2^ Although optimal models of differentiated service delivery remain opaque,^20^, ^21^ maximising their public health impact probably requires systems to be patient-centred and adaptive, with robust quality-improvement processes.^21^ We have tracked engagement with the treatment cascade in Zimbabwe since 2011,^22^ and it is encouraging to see the stepwise increase that has occurred over the past decade.^19^, ^23^ It will be crucial to maintain and build on these improvements^24^ by continuing data-guided programme management,^25^ preventing disengagement among those currently on ART, identifying newly infected female sex workers, and ensuring that the female sex workers at the highest risk get the most support. Innovative surveillance approaches will be needed to identify new places and subpopulations in which transmissions are occurring and to monitor emergence of ART resistance over time. In a systematic review of the engagement of female sex workers with HIV ART treatment across sub-Saharan Africa, treatment engagement has improved in line with that (but less than) in the general population.^2^

The low uptake and suboptimal use of prevention technologies is consistent across Africa, including in non-sex workers;^26^ more work is needed to understand this. Of note, self-reported PrEP use was substantial, but not supported by tenofovir concentrations. Similar results were reported among young women in Kenya, where only 5% of current PrEP recipients had protective drug concentrations.^27^ Other studies also reported this mismatch between reported PrEP use and biomarkers in Kenya.^28^, ^29^ We identified only 36 women among RDS participants who both reported current PrEP use and had a record of receiving a PrEP prescription from the programme in the 3 months before tenofovir diphosphate concentrations were measured; 70% of these had tenofovir diphosphate concentrations greater than 350 fmol/punch but none above 700 fmol/punch. Additionally, we found consistent condom use (as measured through presence of Y chromosome or *N gonorrhea*) was uncommon, with about 40% of the rates that were self-reported. In Asia, the 100% Condom Programmes did result in important incidence declines (although self-reports of condom use were not verified, rates of both STI and HIV incidence declined).^30^, ^31^ Here, we defined inconsistent condom use as any condomless sex in the preceding month through questionnaire and biomarkers and regardless of partner type, which could be more stringent than required to reduce HIV acquisition risk. Categorising partners of sex workers as boyfriends or clients in Zimbabwe is problematic,^32^ with partners transitioning between states potentially affecting consistent condom use. Suboptimal condom use in Zimbabwe could reflect lower levels of social cohesion among female sex workers, where programmes supporting community empowerment started later^33^ and are less entrenched. Poor condom use could also reflect hostility towards female sex workers (eg, female possession of condoms is routinely used by police in Africa as evidence of sex work).^34^, ^35^

The goal of the AMETHIST intervention was to bring about a step change in whole population uptake of and engagement with HIV prevention and care among female sex workers in Zimbabwe. We hypothesised that risk-differentiated support led by female sex workers would ensure that those most at risk of HIV acquisition or transmission would be best supported. Our intervention was feasible to implement, and microplanning has now been scaled up across Zimbabwe. The study is the first to show that risk-differentiated community support for female sex workers can improve linkage to treatment at rates approaching virtual elimination of transmission through sex transactions (only 5·8% of female sex workers were at risk of transmission in the intervention group).

The absence of an effect on prevention uptake is also important, including the mismatch between prevention use data from the programme and biomarker-supported survey. This mismatch has important implications for introducing long-acting, injectable PrEP and underscores the need for further research to fully understand barriers to use of prevention technologies, to help to refine intervention approaches and evaluate them. Female sex workers face numerous barriers to uptake of prevention.^36^ Female sex workers in this trial felt that avoiding becoming HIV positive was often out of their control and not a priority given other challenges—including poor mental health, violence, and substance use. It is possible that overall success in HIV treatment has reduced women's fear of acquiring HIV. Although our AMETHIST intervention was risk-differentiated, we need to understand female sex workers’ risk of HIV acquisition and broader health outcomes more holistically, and how these differ across female sex workers’ life course and their diverse identities and lived experiences.

### Contributors

### Equitable partnership declaration

### Data sharing

Researchers carrying out studies with clearly described objectives can request access to data, including individual de-identified participant data and a data dictionary used in this study through the corresponding author. The request will be considered by the trial investigators. Prespecified data will be made available subject to a written proposal and a signed data sharing agreement.

## Declaration of interests

We declare no competing interests.

## Supplementary Materials

Shona translation of the abstract

Ndebele translation of the abstract

Supplementary appendix 3

Equitable Partnership Declaration
