# Supplementary material for: A risk-differentiated, community-led intervention to strengthen uptake and engagement with HIV prevention and care cascades among female sex workers in Zimbabwe (AMETHIST): a cluster randomised trial
Source: Lancet Glob Health. 2024 Aug 14;12(9):e1424–35. doi: 10.1016/S2214-109X(24)00235-3 (PMC11345450; doi:10.1016/S2214-109X(24)00235-3)
Supplement: Shona translation of the abstract [file mmc1.pdf]

THE LANCET
Global Health

## Supplementary appendix 1

This translation in Shona was submitted by the authors and we reproduce it as supplied. It has not been peer reviewed. *The Lancet*'s editorial processes have only been applied to the original in English, which should serve as reference for this manuscript.

Shanduro iyi muShona yakatumirwa nevanyori uye tinoiburitsa sekupihwa kwatakaitwa. Huye hayina kuongororwa nevezera. Maitiro ekupepeta e Lancet angoshandiswa chete kugwaro rekutanga, iro rinofanira kushanda seumboo hwechinyorwa.

Supplement to: Cowan FM, Machingura F, Ali MS, et al. A risk-differentiated, community-led intervention to strengthen uptake and engagement with HIV prevention and care cascades among female sex workers in Zimbabwe (AMETHIST): a cluster randomised trial. *Lancet Glob Health* 2024; **12:** e1424–35.

**Pfupiso**

**Nhanganyaya** Vanhukadzi vanotengesa bonde vanonetsekana nedambudziko reHIV zvakanynaya kana tichienzanisa nevamwewo vanhukadzi. Chinangwa chetsvakurudzo ino chaive chekuedza kuona kuti rutsigiro rwunowaniswa vanhukadzi ava nevamwe vavo (peers) rwune charwunoita here kuvanhukadzi ava pakuderedza njodzi yekubatira kana kutapurira HIV nenzira yebonde, muZimbabwe.

**Maitirwo** Mutsvakurudzo iyi, makiriniki 22 anobatsira vanhukadzi vanotengesa bonde muZimbabwe akagoverwa 1:1 nenzira inoita kuti pave nekuenzana pakugoverana. 11 akaramba achingoita zvaagara achiita uye mamwe 11 akawaniswazve chirongwa cheAMETHIST. Zvagara zvichingowaniswa zvaisanganisira kuongororwa HIV, kupihwa mapiritsi anoderedza njodzi yekubatira HIV (PrEP), kurudziro yekuenda "pachirongwa" chehurumende, nzira dzekudzivirira pamuviri, makondomu, kurapwa zvirwere zvepabonde, dzidziso yehutano, mazano ezvemutemo uye rutsigiro kubva kune vamwe vanotengesawo bonde. AMETHIST yakawedzera zvirongwa zvinoitisa mari zvaitungamirirwa nevanhukadzi vanotengesa bonde, zvaienderana nekuti njodzi yekubatira HIV yemunhu yakamira sei uyezve, mapoka ekupana rutsigiro rwakasiyana-siyana. Vanhukadzi vose <vane makore 18 zvichikwira> vakambotengesa bonde mumazuva 30 apfuura uye vaigara kana kushanda muzvimbo dzaiitirwa tsvakurudzo, vaikwanisa kupinda mutsvakurudzo iyi. Chikamu chetsvakurudzo hachina kuvanzwa kune avo vaiwanisa zvirongwa asi chakavanzwa kune vaiita tsvakurudzo nevaiongorora ropa. Mushure memwedzi 28, ongororo yepamapoka evanhu vanozivana (RDS survey) yakaitwa nevanhukadzi vanotengesa bonde, vanogara pedyo nekiriniki imwe neimwe. Ongororo iyi yaitarisa dozvo guru retsvakurudzo, iro raiva rekuda kuona kuti chikamu chevanhukadzi vanotengesa bonde chaive nenjodzi yekutapurira HIV (zvichireva, vairarama neHIV, vainge vasiri kudaira "mushonga" (maARV) uye vainge vasiri kushandisa makondomu nguva dzose) kana njodzi yekubatira HIV (zvichireva, vainge vasina HIV asi vasiri kushandisa makondomu kana mapiritsi anoderedza mukana wekubatira HIV (PrEP)) chainge chakakura sei. Tinoratidza zvikamu zvevanhukadzi vanotengesa bonde zveavo vakapinda mutsakurudzo zvaive panjodzi yekutapurira kana kubatira HIV. Tsanangudzo dzedu dzainge dzakatarwa nechekare, dzichitarisawo nzira yakaitwa nayo ongororo uye mazera evakapida mutsvagurudzo. Tsvagurudzo yakanyoreswa kuPan African Clinical Trials Registry, PACTR202007818077777.

**Zvakabuda**

Chirongwa chekuwanisa AMETHIST chakatanga musi wa15 May 2019 uye humboo hwakaunganidzwa kubva 1 June 2019. Ongororo yepamapoka evanhu vanozivana (RDS survey) yakaitwa kubva musi wa18 October kusvika 13 December 2021 nevanhukadzi 2137 vaive muchikamu chisina AMETHIST (makiriniki 11) uye vanhukadzi 2131 vaiwaniswa chirongwa cheAMETHIST (makiriniki 11), mushure mekunge tabvisa vakaparuriswa ongororo (vanoita 132) nevanhukadzi vainge vasina humwe humboo hwakakosha (vanoita 44). 1973 (46·2%) vevanhukadzi 4268 vakapinda muongororo vainge vari kurarama neHIV. Pavanhukadzi ava, 863 (93·5%; pachitariswa maitirwo eRDS) pa931 vaiwaniswa chirongwa cheAMETHIST uye 927 (88·8%) pa1042 vaisawaniswa AMETHIST vaive nehutachiona hwakaderera muropa ravo. Vanhukadzi 287 (22·4%) pa1200 vainge vasina HIV vaiwaniswa chirongwa cheAMETHIST uye 194 (15·7%) pa1096 vaisawaniswa AMETHIST vakatiudza kuti vainge vari kunwa mapiritsi anoderedza mukana wekubatira HIV (PrEP), uye vaviri chete (0·4%) pa569 vainge vane humboo hwekunwa mapiritsi aya muropa ravo inonzi iyo *protective plasma intra-erythrocytic tenofovir diphosphate concentration* spots (>700 fmol/dried blood punch). Hatina kuona humboo hwekuti chirongwa cheAMETHIST chakashanda kana takatarisa donzvo guru retsvakurudzo, iro rekuedza kuderedza mukana wekutapurira uye kubatira HIV (chikamu cheAMETHIST n=1156/2131, pachitariswa maitirwo eRDS 55·3%; chikamu chaisawaniswa AMETHIST n=1104/2137, pachitariswa maitirwo eRDS 52·7%; musiyano pachitariswa zera –0·9%, 95% CI –5·7% to 3·9%, p=0·70). Pazviwewo zvaitariswa patsvakurudzo iyi, chikamu chevanhukadzi vairarama neHIV vane njodzi yekutapurira HIV chainge chakaderera kwazvo, uye njodzi iyi yakaderedzwa

zvakanyanya muchikamu cheAMETHIST (n=63/931, pachitariswa maitirwo eRDS 5·8%) tichienzanisa nechikamu chaisawaniswa AMETHIST (103/1041, 10·4%), paine musiyano pachitariswa zera we –5·5% (95% CI –8·2% to –2·9%, p=0·0003). Njodzi yekubatira HIV pavanhukadzi vaisararama neHIV yainge yakafanana tichienzanisa chikamu cheAMETHIST (n=1093/1200, pachitariswa maitirwo eRDS 92·1%) nechikamu chaisawaniswa AMETHIST (1001/1096, 92·2%), paine musiyano pachitariswa zera we –0·6% (95% CI –4·6 to 3·4, p=0·74).

**Zvazvinoreva** Chirongwa cheAMETHIST hachina kubatsiridza panjodzi yekutapurira kana kubatira HIV. Hutachiona hwemuropa hwainge hwakaderera zvikuru muvanhukadzi vairarama neHIV uye hwakaita sekuti hwakaderedzwa zvakare nechirongwa cheAMETHIST, izvo zvinoratidza sekuti zvikamu zvevanhu vari panjodzi yekubatira HIV, uyezve zvisingagari panzvimbo imwechete, zvinokwanisa kunyatsonwa mishonga yekuderedza HIV nemazvo uyezve sezvinotarisirwa. Zvakakosha kuti parambe pachiwaniswa mishonga yekuderedza HIV uyezve pamutsiridzwe nekusimbisa nzira dzekuidzivirira.
