# Supplementary material for: A risk-differentiated, community-led intervention to strengthen uptake and engagement with HIV prevention and care cascades among female sex workers in Zimbabwe (AMETHIST): a cluster randomised trial
Source: Lancet Glob Health. 2024 Aug 14;12(9):e1424–35. doi: 10.1016/S2214-109X(24)00235-3 (PMC11345450; doi:10.1016/S2214-109X(24)00235-3)
Supplement: Ndebele translation of the abstract [file mmc2.pdf]

THE LANCET
Global Health

Supplementary appendix 2

This translation in Ndebele was submitted by the authors and we reproduce it as supplied. It has not been peer reviewed. *The Lancet*'s editorial processes have only been applied to the original in English, which should serve as reference for this manuscript.

Lokhu kuntshintshwa kolimi [NDEBELE] kuthunyelwe ngaba lobi njalo senza kakutsha njengokuthunyelwa. Akukabhekisiswa ngamaqembu abheka imbhalo. Ukucutshungulwa kwemibhalo okwenziwa ngabe Lancet kwenziwe kuphela kowesilungu [English] yiwo okufanele usebenze njengenkomba yalo mbhalo.

Supplement to: Cowan FM, Machingura F, Ali MS, et al. A risk-differentiated, community-led intervention to strengthen uptake and engagement with HIV prevention and care cascades among female sex workers in Zimbabwe (AMETHIST): a cluster randomised trial. *Lancet Glob Health* 2024; **12:** e1424–35.

Isifinyezo

Ukusungula Abesifazana abathengisa ucansi bahlutshwa ludubo lweHIV kakhulu nxa sibalinganisa labanye abesifazana. Injongo yoxwayisiso lolu yayingeyokuzama ukubona ukuthi usekelo olunikezwa abesifazana abathengisa ucansi ngontanga (peers) kukhona olukwenzayo kwabesifazana laba ekwehliseni ingozi yokuthola kumbe ukuthelela iHIV emacansini kwelase Zimbabwe.

Indlela yokwenza Kuxwayisiso lolu, amakilinika angu 22 ancedisa abesifazana abathengisa ucansi atholakala ezibhedlela kumbe emakilinika kahulumende eZimbabwe anikezwa ngendlela eyenza kube lokulingana ekwabeleni, amakilinika angu 11 aqhubeka ngenhlelo ezihlala zinikezwa amanye angu 11 angena kuhlelo lweAMETHIST. Inhlelo ezihlala zinikezwa zihlanganisela ukuhlolwa iHIV, ukunikezwa amaphilisi ayehlisa ingozi yokuthola iHIV (PrEP), ukudluliselwa ukuyangena ehlelweni lwe antiretroviral therapy (ART), indlela zokuvikela ukuzithwala, amakhondomu, ukwelatshwa kwemikhuhlane yemacansini, imfundiso yempilakahle, ukucebiswa ngokomthetho, njalo usekelo oluvela kwabanye ontanga. I AMETHIST yengeza inhlelo zokuthola imali ezazithunganyelwa ngontanga ezihlelwe zikhangela ingozi umuntu akiyo njalo lamaqembu okuzisiza. Abesifazana bonke (abaleminyaka 18 kusiya phambili) abathengise ucansi phakathi kwamalanga angamatshumi amathathu adluleyo (30 days) njalo besebenzela kumbe behlala endaweni eyayisenzelwa uxwayisiso babefanele bangene kuxwayisiso. Isimo sohlelo kasizange sisithwe kulabo ababenikeza inhlelo kodwa sasithwa kulabo ababesenza uxwayisiso lezisebenzi zase laboratory. Ngemva kwenyanga ezingamatshumi amabili latshiya ngalo mbili (28), kwenziwa inhlolisiso kunani labesifazana abathengisa ucansi ekilinika inye ngayinye, eyayikhangela impumela yoxwayisiso yakuqala, eyayikhangela isixuku sabesifazana abathengisa ucansi kulabo abangena kule nhlolisiso abasengozini yokuthelela iHIV (njengokuthi, ababele HIV, abalenani legcikwane elinengi njalo bengasebenzisi amakhondomu sikhathi sonke) kumbe abasengozini yokuthola iHIV (njengokuthi, abangela HIV njalo bengasebenzisi amakhondomu sikhathi sonke kumbe bengekho kuPrEP). Sibika izixuku zabesifazana abathengisa ucansi kunani elangena kunhlolisiso kulabo abasengozini yokuthelela kumbe ukuthola iHIV. Ukuhlaziya kwakucacisiwe, kukhangelwa indlela okwenziwa ngayo inhlolisiso njalo leminyaka yalabo abangena kuxwayisiso. Uxwayisiso lwabhaliswa ePan African Clinical Trials Registry, PACTR202007818077777.

Okwatholakalayo Uhlelo lwe AMETHIST lwaqala mhlaka 15 Nkwenkwezi (May), 2019, njalo ulwazi lwabuthaniswa kusukela mhlaka 1 Nhlangula (June), 2019. Inhlolisiso yamaqembu azanayo (RDS survey) yenziwa kusukela mhlaka 18 Mfumfu (October) kusiya fika mhlaka 13 Mpalakazi (December) 2021, labesifazana abangu 2137 kuhlelo oluhlala lunikezwa (amakilinika angu 11) njalo labesifazana abangu 2131 kuhlelo lweAMETHIST (amakilinika angu 11) ngemva kokukhipha abasungula inhlolisiso (abayi 132) njalo labesifazana ababengela lwazi oluqakathekileyo (abayi 44). 1973 (46·2%) yabesifazana abayi 4268 abangena kunhlolisiso babephila le HIV; kulabo, abayi 863(93·5%; kukhangelwa ukwenziwa kwe RDS) sibakhipha kwabesifazana abayi 931 ababekuhlelo lwe AMETHIST njalo abayo 927 (88·8%) sibakhipha ku 1042 yabesifazana ababekuhlelo oluhlala lunikezwa babelegcikwane elilutshwane egazini labo. Abesifazana abayi 287(22·4%) sikhipha ku 1200 yabangelayo iHIV kuhlelo lweAMETHIST njalo abayi 1096 kuhlelo oluhlala lunikezwa babika ukuthi bathatha imithi yokuvikela ukuthi bangatholi iHIV (iPrEP) okwamanje, kulabo, ababili kuphela (0.4%) sikhipha ku 569 babelezitshengiselo zokunatha amaphilisi la egazini labo ethiwa yi *protective plasma intra-erythrocytic tenofovir diphosphate concentrations* eyahlolwa egazini labo elomileyo (>700 fmol/dried blood punch). Asibonanga ukuthi uhlelo lwe AMETHIST lwasebenza sikhangela impumela yoxwayisiso yakuqala eyokuzama ukwehlisa amathuba okuthelela kumbe ukuthola iHIV (uhlelo lwe AMETHIST n=1156/2131, kukhangelwa ukwenziwa kwe RDS 55·3%; uhlelo oluhlala lunikezwa n=1104/2137, sikhangela ukwenziwa kwe RDS 52·7%; umtshiyano kukhangelwa iminyaka difference –0·9%, 95% CI –5·7% to 3·9%, p=0·70). Impumela yoxwayisiso eyesibili, isixuku sabesifazana abaphila le HIV abasengozini yokuthelela abanye yayiphansi njalo yehla kakhulu kuhlelo lwe AMETHIST (n=63/931, sikhangela ukwenziwa kwe RDS 5·8%) siqathanisa leqembu elikuhlelo oluhlala lukhona

(103/1041, 10·4%), kulomehluko kukhangelwa iminyaka owe 5·5% (95% CI –8·2% to –2·9%, p=0·0003). Ingozi yokuthola I HIV kwabesifazana abangela HIV yayifana kuhlelo lwe AMETHIST (n=1093/1200, kukhangelwa ukwenziwa kwe RDS 92·1%) lakuqembu elikuhlelo oluhlala lukhona (1001/1096, 92·2%), kulomehluko kukhangelwa iminyaka –0·6% (95% CI –4·6 to 3·4, p=0·74).

Ingcazelo Kwakungela nzuzo kuhlelo lwe AMETHIST kungozi yokuthelela kumbe ukuthola iHIV. Igcikwane latholakala lehlile egazini labesifazana abaphila le HIV njalo kwakhanya kuthuthukiswa luhlelo lweAMETHIST, lokhu kutshengisa abantu abasengozini yokuthola I HIV lalabo abangahlali ndawonye bangenelisa ukunatha imithi yokwehlisa iHIV (ART) njalo behlale beyinatha ngemfanelo. Kuqakathekile ukuthi imithi yokwehlisa iHIV iqhubeke itholakala njalo kuvuselelwe lokuqinisa indlela zokuvikela.
