# Supplementary material for: A risk-differentiated, community-led intervention to strengthen uptake and engagement with HIV prevention and care cascades among female sex workers in Zimbabwe (AMETHIST): a cluster randomised trial
Source: Lancet Glob Health. 2024 Aug 14;12(9):e1424–35. doi: 10.1016/S2214-109X(24)00235-3 (PMC11345450; doi:10.1016/S2214-109X(24)00235-3)
Supplement: Supplementary appendix 3 [file mmc3.pdf]

THE LANCET
Global Health

Supplementary appendix 3

This appendix formed part of the original submission and has been peer reviewed. We post it as supplied by the authors.

Supplement to: Cowan FM, Machingura F, Ali MS, et al. A risk-differentiated, community-led intervention to strengthen uptake and engagement with HIV prevention and care cascades among female sex workers in Zimbabwe (AMETHIST): a cluster randomised trial. *Lancet Glob Health* 2024; **12:** e1424–35.

APPENDICES

1. AMETHIST Trial methods in full

2. Trial randomisation procedure

3. Process Evaluation Framework

4. Structured description of the intervention.

5. Primary outcome algorithm

6. List of condom use questions

7. Statistical analysis methods

8. RDS diagnostics methods and results

9. R Code for primary outcome

10. Sensitivity analyses

APPENDIX 1: FULL TRIAL METHODS

Methods

Study design and participants

The AMEHTIST trial was a cluster randomised trial nested within Zimbabwe's nationally scaled programme for sex workers, the Key Populations (KP) Programme (formerly known as "Sisters"))run on behalf of the Ministry of Health and Child Care and National AIDS Council, detailed elsewhere.[6] Twenty-two clusters were purposively selected from 57 KP programme sites across Zimbabwe to reflect a range of settings and be of adequate size (Appendix pages 2-3). Clusters were randomised 1:1 on January 29th 2019 using restricted randomisation (using programme data from 2017). An endline survey was conducted 18th October – 13th December 2021 after 28 months of intervention implementation in all 22 clusters using respondent driven sampling (RDS). FSW were eligible if they had exchanged sex for money in the previous 30 days, were aged 18 years or older, and had been living or working for at least 1 month in the cluster where they were interviewed. It was not possible to mask intervention status from programme implementers, but survey teams were blinded to intervention status and all survey teams ran surveys in both intervention and control communities. Laboratory staff were unaware of intervention allocation. We nested a mixed methods process evaluation within the trial, following an adapted version of the MRC Guidance for Process Evaluations of Complex Interventions[7] to assess the fidelity, feasibility, acceptability, coverage and quality of intervention delivery, see Appendix page 4 for details and tools.

All cis gender women who sell sex and who live and or work within trial clusters were eligible to access the KP programme +/- the AMETHIST intervention (intervention arm). Written consent was not required for programme participation. Written informed consent, in English, Shona, or Ndebele, was obtained for survey participation before interviews were done and biological samples were taken.

Ethics approval was obtained from the Medical Research Council of Zimbabwe (MRCZ/A/2559), Liverpool School of Tropical Medicine (19-115RS) and the London School of Hygiene & Tropical Medicine (19123).

Procedures

Usual care group:

The KP programme provided HIV services as outlined in the TiDIER framework (Appendix page 5) including comprehensive sexual and reproductive health services, HIV testing and PrEP following WHO guidelines.[8,9] Women requiring HIV care were referred to government services. Activities were supported by trained peer educators. Services were provided at drop-in centres based in primary-care clinics on the same day each week. Outreach worker supervisors (ORWs) (salaried social workers)) met with peer educators as a group once a month per site.

Intervention group:

The AMETHIST intervention was implemented in addition to usual care activities. The AMETHIST intervention and its mechanisms of action are shown in the TiDIER framework (Appendix page 5) which has been updated since published in our protocol paper [6] to reflect early modifications. The intervention comprised two components, FSW peer-led microplanning (systematic, risk-differentiated peer-led community-based support for all sex workers in a cluster)[10,11] and SHG for sex workers enrolled in microplanning. We aimed to enrol around 50% of FSW into SHGs in each cluster by the end of the trial. Peer-microplanners took responsibility for a geographic hotspot where sex work activity

was identified, attempting to recruit all FSW working there (50-80 per peer-microplanner). They assessed risk of FSWFSW in their caseloads every three months using a simple risk score and tailored level of follow up according to level of risk. S FSW at high risk were seen weekly, those at moderate risk twice a month and those at low risk once a month. Information on HIV status was not collected by peer-microplanners and the community support they delivered was HIV status neutral. They collected data from their caseload, including risk assessments, and recorded meetings with FSW. These data were used to guide their discussions with ORWs. In the intervention arm, ORW met with peer-microplanners each week to review their caseload with them and discuss challenges of risk-differentiated support over the previous week, then planned activities for the following week.

SHG aimed to build social cohesion as well as psychological and potentially financial resilience among members and the wider sex work community. ORW supported peer-microplanners to establish and maintain SHG. The intention was for each microplanner to establish two SHG for 13-15 FSW during the trial.

Sampling for outcomes strategy

An endline survey was done in all 22 sites between October 18th to December 13th, 2021 after 28 months of intervention. Survey participants were recruited using RDS. In each cluster, we used geographical and social mapping to select six women per site as "seeds", representing diverse ages, types of sex work, and geographical locations. We interviewed FSW, gave each two coupons to distribute to peers over 2 weeks, and read them a sample recruitment script. Women who received a coupon could attend an interview, and on interview completion were given two coupons for their peers. In all clusters, this process was repeated until recruitment of at least 200 women. Participants received US$5 and a further US$2 for each woman she recruited. A coupon management system ensured coupons were genuine and minimised repeat participation.

The questionnaire was self-administered using audio computer assisted survey instrument (ACASI) to minimise social desirability bias[12] and included questions on demographics, sex work, sexual behaviour, condom use, HIV testing history, ART use, stigma, experience of violence, quality of life, mental health, general health, relationships with other sex workers, contact with peer educators / peer-microplanners, participation in SHG and use of sexual and reproductive health services including through the KP programme. Data were collected on tablet computers and uploaded to a database daily. To estimate RDS-II weights, we asked each participant how many FSW older than 18 years they knew living in the site whom they had seen in the past month and would consider recruiting to the study.

All women had a finger prick sample collected for testing for HIV infection and syphilis as point of care tests and received results on site. Participants also had two dried blood spot samples (DBS) collected for HIV viral load testing and PrEP level testing. They were asked to provide two self-administered vaginal swabs to be tested for STIs (Neisseria gonorrhoea, Chlamydia trachomatis and Trichomonas vaginalis) and, if reporting consistent condom use for the last 2 weeks, for Y chromosome. The results of viral load were made available to women within 4 weeks of the survey. Results of STI tests were made available through the KP clinic within 4 weeks of the survey, where free treatment is available.

Finger prick blood samples taken for HIV were tested on site according to the Zimbabwe National HIV testing algorithm with samples tested in series. The syphilis sample was tested using DPP® Syphilis Screen & Confirm Assay (Chembio Diagnostic Systems, Inc. New York, USA), a near patient test for both Rapid Plasma Reagin (RPR) and Treponema Pallidum Haemagglutination Assay (TPHA).DBS samples were air dried on filter papers and stored at room temperature until they transported every 7 days, to the Zvitambo Laboratory (Harare, Zimbabwe) for processing and storage.

Women who tested positive for HIV had a DBS sample tested to quantify viral load using NucliSENS EasyQ HIV-1 version 2.0 (bioMerieux, Marcy L'Etoile, France). For HIV negative women who reported currently taking PrEP, DBS samples were sent to University of Cape Town for Tenofovir-diphosphate (TDF-DP) levels (protective levels of PrEP are defined as TDF-DP ≥ 700 fmol/dDBS punch and partially protective if between 350-700fmol/punch).

Vaginal samples were kept refrigerated (2–8°C) and transported to the laboratory within 7 days. STI samples were tested by the Newlands Clinic using Allplex™ STI Essential Assay Q (Seegene Inc Seoul, Republic of Korea). Y chromosome samples were tested by National University of Science and Technology using the Quantifiler™ Trio DNA Quantification Kit (Life Technologies LTD, Warrington UK).

Outcomes

Our primary composite outcome was designed to measure the impact of a status neutral intervention across the whole population of FSW regardless of HIV status. In our protocol paper we justified this approach[6] and discussed a number of complexities for interpretation of the proposed composite outcome, including recognition that we might see different effects among HIV positive and negative participants and so it captures the proportion of all survey participants at risk of either HIV acquisition or transmission after 28 months. The primary outcome is intended to capture the proportion of all FSW participants in the RDS survey who are at risk of either HIV acquisition or transmission after 28 months of intervention. Risk of HIV acquisition is defined as being HIV negative and having any sex in the last month that is not protected by a condom or not having protective levels of PrEP. Women self-reporting consistent condom use were re-categorised as having had any condomless sex if they had Y chromosome or gonorrhoea detected on vaginal sample PCR. Risk of transmission is defined as being HIV positive with a viral load >1000copies/uL and having sex in the last month that is not protected by a condom (as previously defined). The algorithm for allocating women to these four states i) HIV negative and at any risk of acquisition (ii) HIV negative and not at risk of acquisition; or iii) HIV positive and at risk of transmission (iv) or HIV positive and not at risk of transmission, has been published previously [6] and is shown in the Appendix (pages 6-7); Figure A4 number of FSW in each category). The condom use questions used in the survey are shown in Appendix page 8.

In addition to the primary outcome, we report here a pre-specified secondary sub-group analysis, stratifying the main analysis by HIV status. (Appendix pages 21-23) Since this analysis involves stratification by HIV status based on the endline survey we recognise that our separate analyses by HIV status are not randomised comparisons.

Statistical analysis

Our sample-size calculations have been described previously.[6] In our previous trial, k was 0.17 so we estimated sample size using both a k of 0.2 and 0.25. In most scenarios, we estimate 90% power to detect a 30% difference in proportion of FSW at risk of HIV acquisition/transmission between intervention and control arms from 30% to 21%. If k is 0.25 we have 78% power to detect a 30% difference from 30% to 21%. and over 99% power to detect a 50% difference from 30% to 15%.[6]

Statistical analysis followed a prespecified plan (Appendix pages 9-10). We assessed evidence of bias in our operationalisation of RDS by graphically examining the convergence of the primary outcome (Appendix pages 11-18). Analyses were done at the cluster level. We accounted for RDS in our estimates of cluster characteristics with RDS-II weighting. We included all RDS participants except te seed respondents and those missing key outcome data (HIV test, VL, TDF, STI or Y chromosome data)

ie conducted a complete case analysis, regardless of cluster- or individual-level adherence and weighted the results for each woman in each site by the inverse of her reported network size (ie, the number of other women that she could have recruited).[13] The target estimand is the difference in transmission/acquisition risk and is derived using an intention to treat analysis of a cluster randomised trial.

We described sociodemographic characteristics of the recruited sample at endline and report the cluster means and ranges by trial arm after RDS-II weighting. For the outcome analyses, we used an adapted cluster summary approach to estimate risk differences, comparing the adjusted and unadjusted means of the RDS-II-weighted site-specific proportions of the binary outcomes in each trial arm. We adjusted the model for age (pre-specified) with the two-step method to adjust for individual level covariates in the cluster-summary analysis (Appendix pages 9-10).[6] The respondent-driven sampling diagnostics code for the primary analysis was written with the RDS package [19] in R (R Core Team (2023). R version 4.1.3; R Foundation for Statistical Computing, Vienna, Austria.) (Appendix pages 19-20) and shared with the trial data safety and monitoring board.

We undertook five sensitivity analyses relating to the primary outcome: first we dropped one control site from the analysis that implemented microplanning (ie, the cluster experienced contamination); second, we ran the analysis without RDS-2 weighting; third, we ran the analysis among those who attended KP clinic (intended to reflect an 'on treatment' analysis); fourth, we used a successive sampling approach (with different population size at each site) to treat the RDS data; and fifth, we used a cut-off of TDF 350 fmol/punch (plus any detectable level) as protective against HIV acquisition (Appendix page 21-23)

Study Registration

Funding for programmatic intervention was received in 1 April 2018 and for research 1 April 2019 from different funders. A protocol to randomise 22 clusters to intervention or standard of care and assess differences in programmatic outcomes received approval from the Medical Research Council of Zimbabwe on 1 October 2018. Randomisation was conducted on 29th January 2019 and intervention delivery commenced May 2019. With receipt of research funding on 1 April 2019 (for a series of studies including the AMETHIST trial) a new protocol was developed including for the trial including all biobehavioural outcomes described here. This protocol received ethics approval from LSTM on 19th February 2020 (conditional on local MRCZ approval) at which time it was submitted to the Pan African Trials Registry for registration (2nd March 2020). SARS-COV2 lockdowns commenced in southern Africa in March 2020, delaying regulatory permissions for non-SARS-COV2 related studies by several months. Full approval for the amended trial protocol was received from MRCZ 10th June 2020. PACTR registered the trial 2nd July 2020 (PACTR202007818077777). No research data were collected prior to full ethics approval and trial registration.

Role of the funding source

The funder of the study had no role in study design, data collection, analysis, interpretation, or writing of the report. The corresponding author had full access to all data and had final responsibility for the decision to submit for publication.

APPENDIX 2: TRIAL RANDOMISATION PROCEDURE

Cluster definition and selection

Twenty-two clusters were purposively selected from 57 Sisters programme sites across Zimbabwe to reflect a range of settings and be of adequate size. A cluster is defined as the FSW population working in the geographic location (usually a town or business centre) where there is a government health clinic providing dedicated FSW services through the Sisters programme. Trial sites were purposively selected to be reflective of a range of settings, of adequate size to ensure participation of between 175 and 475 FSW annually (based on 2017 programme data) (mean 314; 50% seen for first time) and located at geographic spacing sufficient to ensure that the risk of contamination/spill-over of intervention effect between study clusters through FSW mobility and migration will be minimised.

Randomisation

Twenty-two sites were randomised (1:1) to receive the AMETHIST intervention in addition to standard of care or to continue with the standard of care alone. Randomisation was conducted in January 2019 at a public meeting with key stakeholders, MoHCC, district representatives and representatives of female sex worker community from the 22 sites. It was not possible to blind intervention staff or beneficiaries of services to the intervention allocation.

To minimise baseline imbalance between arms, restricted randomisation was used. Restriction factors included province, number of FSW seen in the Sisters programme in 2017, mean age of first-time attenders, proportion of FSW attending the programme who were aged under 20 years of age, proportion of all attendees aware of HIV status, proportion of all HIV-positive attendees on ART and mean number of visits by attendees.

Restricted randomisation resulted in a good balance of restriction factors between treatment arms. Restricted randomisation generated a list of 705,432 potential combinations from which 999 with the closest balance between arms were selected. Non-investigator attendees were invited to withdraw numbers from a bag to see which one of the 999 combinations was selected.

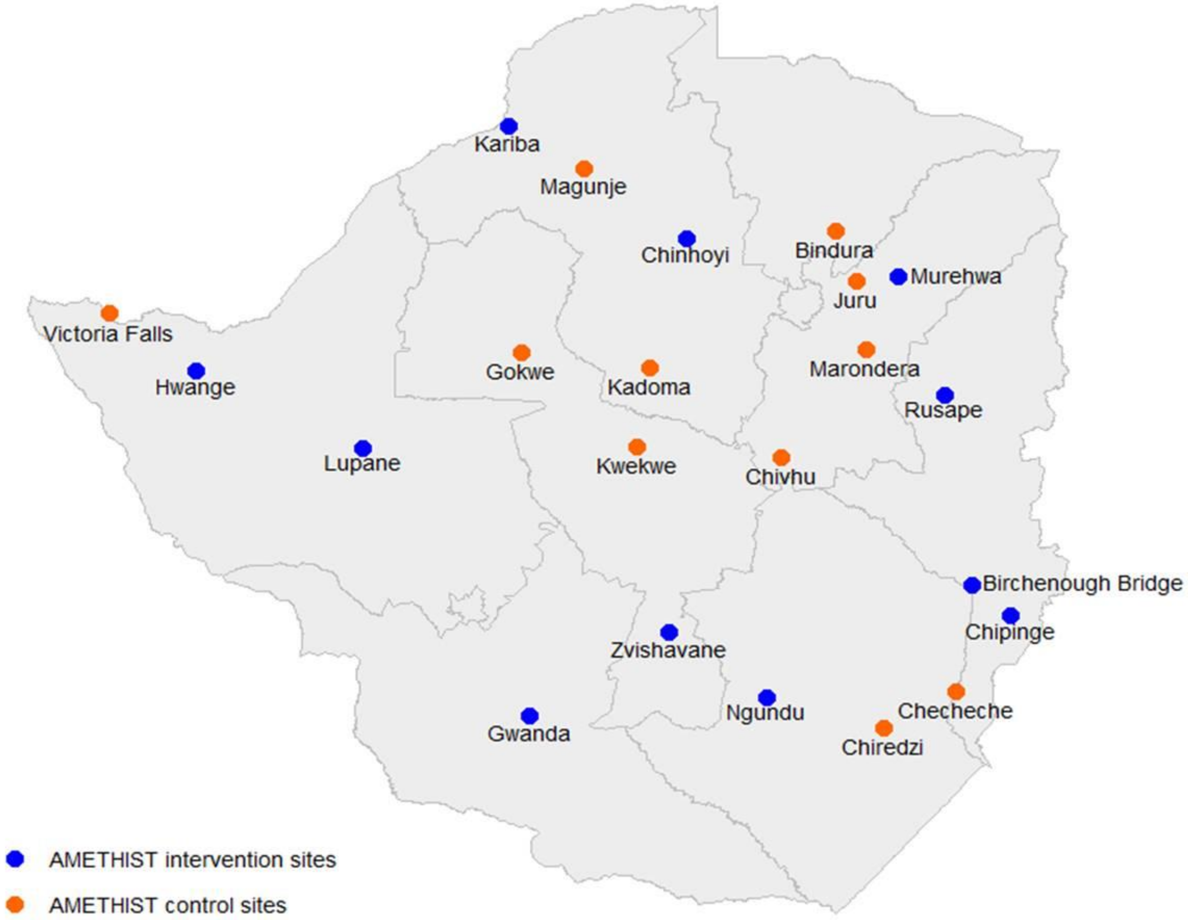

Figure A1: Map showing location of study sites and allocation (colours).

Table A2: Baseline characteristics and balance in restricted randomisation

Characteristics	AMETHIST sites	Usual Care sites
Mean number of FSW seen in the Sisters programme in 2017	348	286
Mean age of first-time attenders	29	30
% of FSW <20 years of age	10	6
% of all attendees aware of HIV status	73	80
% of all positive attendees on ART	52	50
Mean number of visits by attendees	2	2

APPENDIX 2: AMETHIST PROCESS EVALUATION FRAMEWORK AND STRUCTURED DESCRIPTION OF THE INTERVENTION

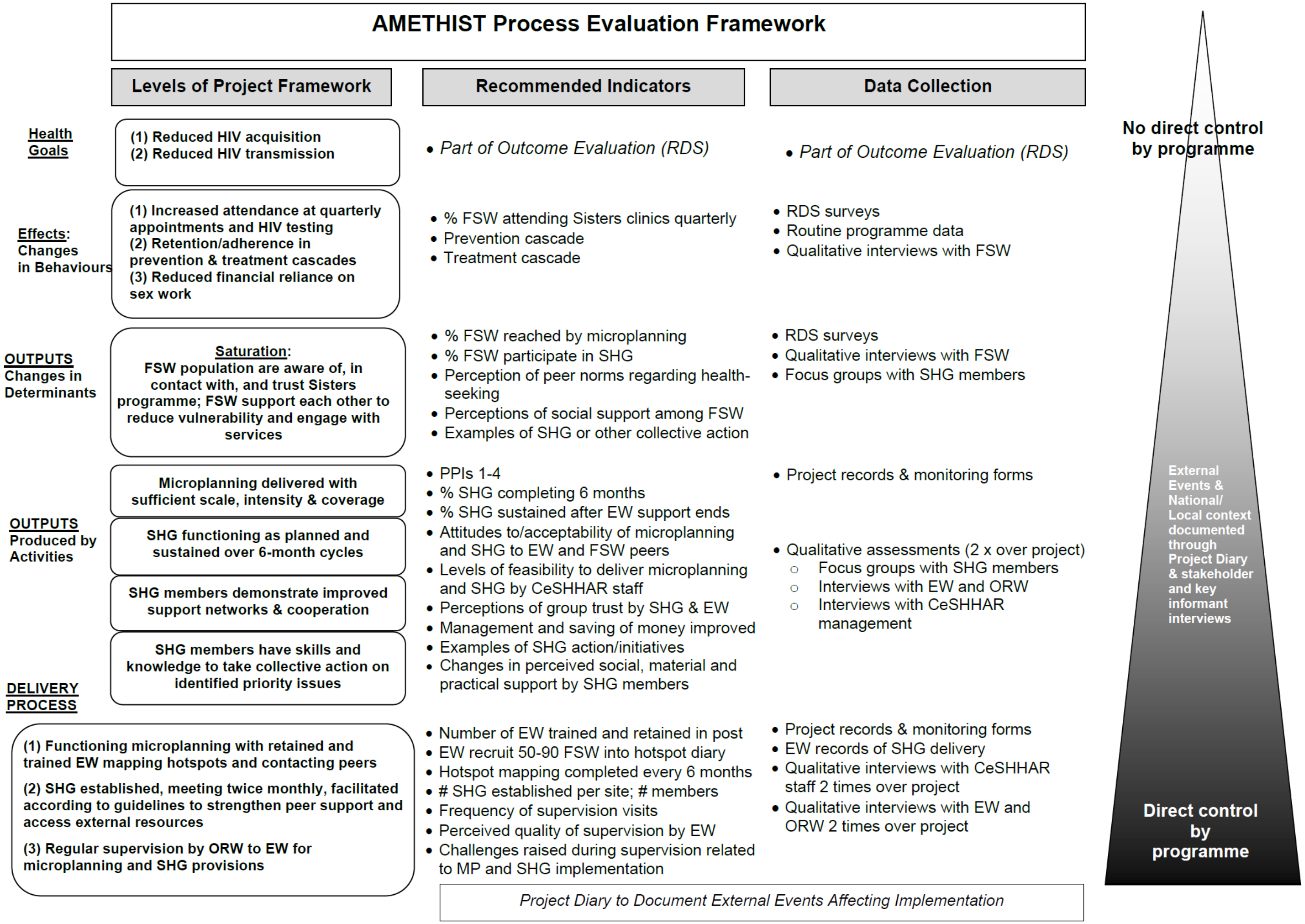

Figure 2: AMETHIST Process Evaluation Framework

Table A3: Updated structured description of the AMETHIST intervention and Standard of Care within which this intervention is set, drawing on TIDIER and Proctor frameworks.

**Brief name**	AMETHIST		Context
	Microplanning	Self help groups	Standard of Care
**Why this intervention now, how will it lead to intended outcomes?**	The Sisters (standard of care) Programme] augemented by i) regular, risk differentiated and structured one to one contact with peers (through microplanning) that nudges [ref] individuals and their networks to reflect on what they need to stay safe; ii) strengthened solidarity [ref] and space to discuss prevention, testing and treatment interventions and negotiate identities and norms (through participation in self-help groups); iii) a conduit, from woman to microplanner to programme, facilitating transfer of information through the system and supporting programmatic response.		The Sisters with a Voice (Sisters) programme operates nationally within 57 primary care clinics and provides comprehensive sexual and reproductive health and HIV prevention services in line with WHO guidelines. Sex workers who test HIV positive are referred to Zimbabwe's HIV treatment programme. Sex workers who become pregnant are referred for prevention of mother to child transmission services.
**What materials are there?**	Microplanning manual, Hotspot diaries and microplanning app to track micoplanning activities. Condoms - with provision tailored to stated need.	Self Help Group manual and registers to record self help group attendance	Sisters Community Mobilisation Manual, Young Sisters Activity pack, Adherence Sisters Support programme manual, condoms as requested
**Who were the actors?**	11 Outreach workers<br>87 Sex worker peer microplanners (reimbursed $50/month)		4 Regional coordinator supervises clinical staff (33 nurses) to provide all clinical services. 36 Outreach workers provide support to 400 peer educators (reimbursed $15/month)
**What did these actors do? What procedures did they follow?**	Outreach workers train microplanners to identify geographic sex work 'hotspots', enumerate local FSW, assess their vulnerability, and tailor support to each FSW's level of social and clinical vulnerability. Each microplanner is responsible ≈50 FSW. Microplanners enrol FSW and provide support according to individual need as defined by a simple vulnerability algorithm.	Each microplanner invites some women in her caseload to join a self help group comprised of 10-15 women who meet twice each month. Each SHG is assisted to identify priorities and receives support towards these. Training is provided to support SHG with their priorities.	**Community and clinic staff** provide Sisters services as described above
**Target for the actions**	All FSWs with around 50% invited to participate in self help group based ontheir vunerability / desire to take part		All FSW
**Tailoring**	Risk is ascertained using a simple score and is reassessed at 3 monthly intervals. FSW at highest risk are seen weekly, those at medium risk twice a month and those at low risk are seen monthly.	None	None
**Target behaviours or behavioural determinants or implementation outcomes**	Peer-led microplanning aims to optimise coverage of services in a given geographic location - and through that the **uptake of and engagement and continuation in HIV prevention and care** (and SRH more broadly), by befriending FSW and thereby developing a trusted relationship with the Sisters programme. At their regular (weekly/bi weekly or monthly) meeting with individual FSW, microplanners follow up on concerns, remind them about clinic appointments/ prescription refills and provide aequate condoms.If they miss appointments they follow them up to ensure they link to services.	FSW community engaged in collective action to improve resilience and reduce vulnerability<br><br>Over time, these groups strengthen programme ownership and community empowerment, to reduce HIV risk and increase uptake of services among FSW and women generally.	Provide comprehensive accessible services for sex workers mobilised using trained peer educators who provide health education and condoms in the community
**How?**	All face 2 face or through whatsapp contacts<br>All activities done with individuals	All acivities done in groups of size 10-15 FSW.	Invidiual or group face 2 face plus follow up phone where able
**Where?**	11 towns around Zimbabwe that provide Sisters services were randomised to the AMETHIST intervention		11 towns where Sisters is implemented around Zimbabwe were radnomised to standard of care
	Microplanners enrol FSW in their geogrpahic hotspot then provide regular follow up in the community at a place which is convenient to FSW (this may include the clinic)	At a community location chosen by the SHG participants	In clinics and hospitals where the Sisters services operate and in the surrounding community
**When and how much**	All microplanners assess the sex workers in their caseload to determine their risk/vulnerability every 3 months using a six point risk score. Sex workers who are assessed as at high risk are seen by the microplanner weekly, those at moderate risk every two weeks and at low risk are seen monthly. Each geographic region is remapped every 6 months to confirm size of sex worker population and optimise the focus of microplanning.	Two times a month	When peer educator meets with individuals or groups on an ad hoc basis to run community mobilisation sesssions or provide access to condoms and condom education

APPENDIX 3: PRIMARY OUTCOME ALGORITHM

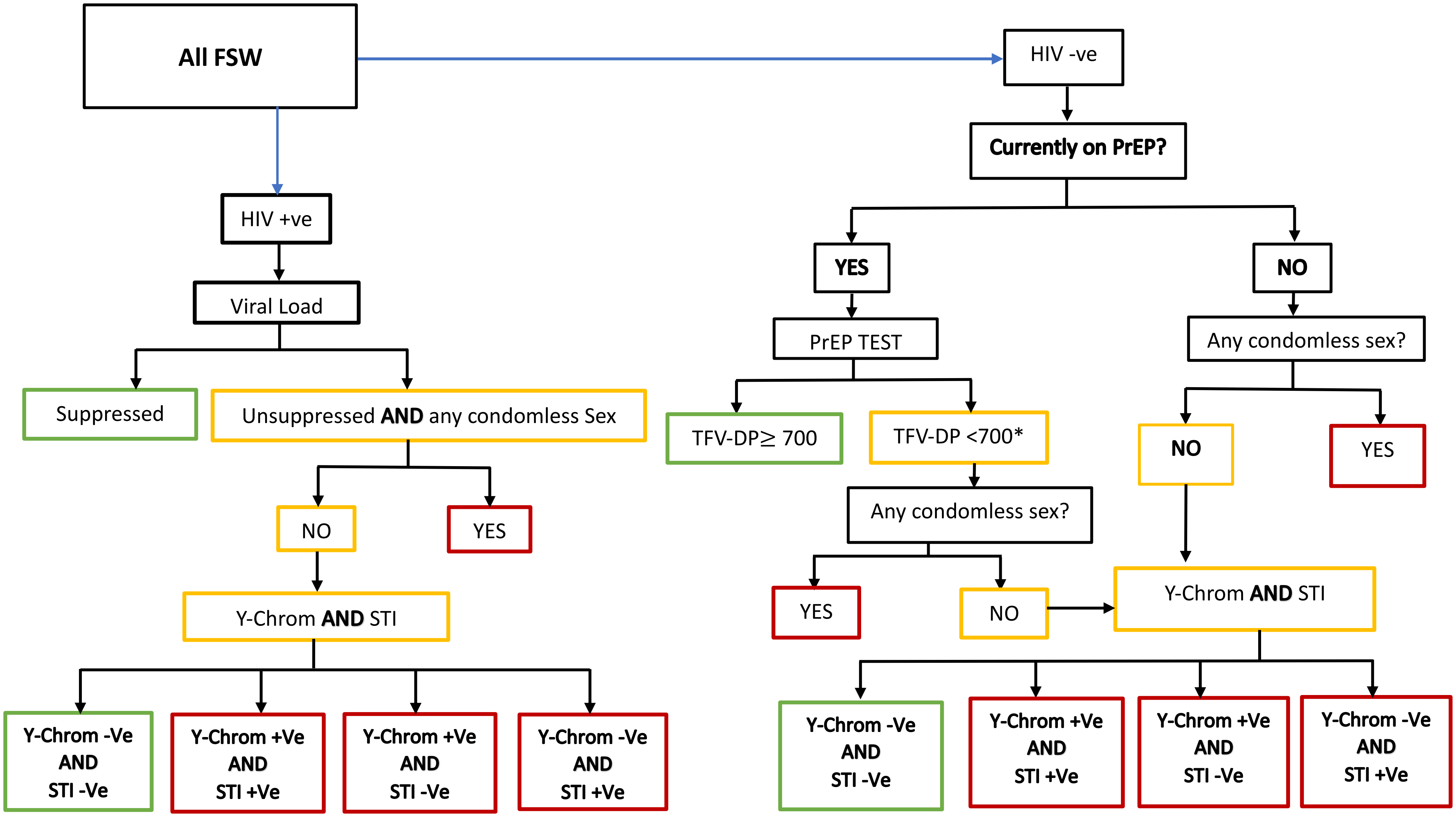

Figure A3: Primary outcome Algorithm. Y-Chromosome test (Y-Chrom), Sexually Transmitted Infections (STI), PrEP (Pre-exposure prophylaxis), Positive (+Ve), and Negative (-Ve). FSW is at risk of transmission (Red) or not at risk of Transmission (Green), Yellow means additional tests are needed to classify them as "at risk" or "not at risk" of HIV transmission/acquisition.

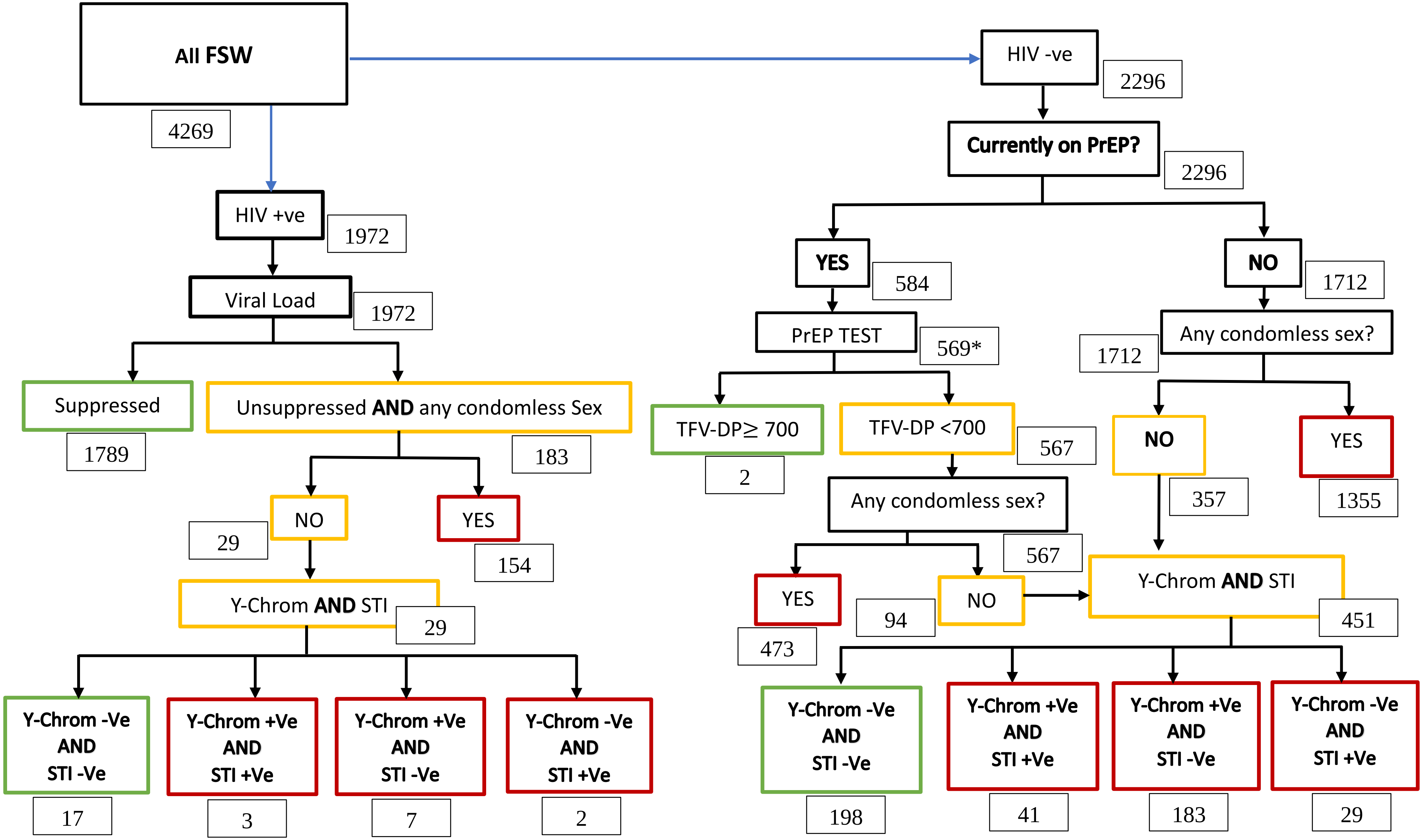

Figure A4: Number of FSWs with different categories of primary outcome algorithm.

**15 participants with missing PrEP test result*

APPENDIX **4: LIST OF CONDOM USE QUESTIONS**

Table A4: List of questions to ascertain consistent condom use

1	Did you use a condom the last time that you had vaginal sex? (I2)	Yes/No	3555 (83.3%) | 713 (16.7%)
2	In the past two weeks, did you use a condom every time you had vaginal sex? (I3)	Yes/No	3248 (80.8%) | 773 (19.2%)
3	Did you use condom the last time you had anal sex (I5)	Yes/No	1460 (81.1%) | 314 (7.4%)
4	In the past two weeks, did you use a condom every time you had anal sex? (I6)	Yes/No	**1384 (76.8%) | 417 (23.2%)**
5	Do you currently have a steady partner (I7) Yes /No<br>If Yes to I7, In the past month, how often did yose condoms with your steady partner? (I10)	Always, most of the time, some of the time rarely, never	1221 (44.3%) | 1534 (55.7%)
6	If yes to I7, The last time you had sex with your steady partner, did you use a condom with him? (I11)	Yes/No	1672 (60.7%) | 1068 (38.8%)
7	In the past month, have there been any times you did not use condoms? (I15)	Yes/No	1958 (45.9%) | 2310 (54.1%)
8	The last time you had sex with a new client, did you use a condom? (I24)	Yes/No	3878 (90.9%) | 390 (9.1%)
9	The last time you had sex with a repeat client, did you use a condom? (I25)	Always, most of the time, some of the time rarely, never	2650 (62.1%) | 1618 (37.9%)
10	In the past month, how often did you use condom with your clients (I26)	Yes/No	2628 (82.4%) | 562 (17.6%)
11	Thinking again about all your clients in the last month, have there been any times when you did not use condoms? (I27)	Yes/No	2014 (76%) | 636(14.9%)
12	With how many clients in the last month did you not use a condom (I28)	Numeric (0, +)	3632 (85.1%) | 636 (14.9%)
13	How many men, other than clients, did you had sex with but did not use a condom in the last month (I29)	Numeric (0, +)	2027 (47.5%) | 2241(52.5%)
**Summary**			
**Total reporting any condomless sex**		n/N	3403/4268 (79.7%)

APPENDIX 5: STATISTICAL ANALYSIS METHODS

The design and proposed analysis strategy for the trial was published before data collection. [1]. We then developed a more detailed statistical analysis plan and discussed and agreed this with the DSMB.

Our analysis was based on the intention-to-treat principle. To account for clustering in the data, data from individuals were summarised for each cluster. We calculated cluster summary means using the RDS-2 methodology developed by Volz and Heckathorn in which individuals are weighted with proportion to 1/self-reported out-degree. [2] To estimate network degree, we used the number of sex workers a participant reported knowing who 18 years were at least old, lived at the site, and who the participant would consider recruiting to the study.

For the crude/unadjusted analysis, we fitted a linear regression model on the RDS-weighted cluster summaries of the outcomes, with a treatment dummy as regressors. We then extracted confidence intervals and the p-value and for the coefficient of the treatment dummy.

To address the possibility of confounding from chance imbalance, we conducted an adjusted analysis using the ‘two step’ method of Hayes and Moulton. [3] Age was specified a priori and included as potential confounder that could affect the outcome but were not on the causal pathway. Other factors would have been included if they appeared imbalanced.[1] But after preliminary exploration of the data no further adjustments were planned. A potentially important imbalance in HIV status was noted between the arms. There was concern both that adjusting for this factor would be necessary to account for possible baseline imbalance in HIV status, but also some concern that HIV status at endline might be partly a factor on the causal pathway between the intervention and the primary outcome. We had already planned to conduct an analysis stratified by HIV status as the main secondary analysis of the data - and this analysis showed some evidence (not statistically significant interaction) that the effect of the intervention differed by individual level HIV status. Ultimately, we decided to conduct a sensitivity analysis of the main primary analysis adjusting for individual level HIV status and be appropriately cautious in our interpretation of this effect.

Approach to generate adjusted, RDS-weighted analysis:

First, an individual-level logistic regression model was fitted with the primary outcome as the dependent variable, and age (continuous) as independent variable. This is shown for the ith individual in the jth intervention arm, below:

$$\log\left(\frac{\mathrm{p}ij}{1-\mathrm{p}ij}\right) = a + B1 * \mathrm{Age}\ ij$$

Where $B1$ is vector of coefficient for the indicator variable associated with age.

This fitted model model was used to generate the predicted probabilities for each woman using the 'predict' function in R. Then, for each cluster, we calculated the weighted arithmetic mean of the predicted probabilities, weighted by the inverse of the network degree to account for the respondent driven sampling (RDS II). The weighted mean of the predicted probabilities was interpreted as the RDS-II-weighted predicted prevalence for each cluster.

Second, we calculated the 'residual' risk difference by subtracting the RDS-II-weighted predicted prevalence (Step 1) from the RDS-II weighted observed prevalence (unadjusted) in each cluster. The, we fitted a linear regression model on the residuals to calculate the adjusted risk difference:

$$\mathrm{RD}j = a + B1 * \mathrm{Intervention}\mathrm{j}$$

Where $R\mathrm{D}j$ is the residual for the jth intervention arm; $B1$ is the risk difference and primary effect estimate. The strength of evidence against the null was assessed using the confidence interval.

APPENDIX 6: RDS DIAGNOSTICS METHODS AND RESULTS

We recruited survey participants in our trial across 22 sites at endline through respondent driven sampling. We have described in the protocol paper the justification for how we treat these data in line with CONSORT principles and the statistical analysis approach, RDS II weighted. [1]

RDS II estimation is based upon a random walk model upon which makes a number of assumptions about the sampling process. These include i) participants are able to accurately report their network size and that they recruit randomly from within this network; ii) seed characteristics do not bias the final estimates; iii) the whole social network of female sex workers is connected at each site; and iv) social ties are reciprocated (recruitees also know their recruiters). The model also assumes with - replacement sampling - that is, that participants can be recruited more than once, which is not the case in practice. However, the extent to which some of these assumptions might be biasing study findings can be tested using information collected in the survey. We conducted a set of recommended RDS diagnostics for each site, to critically appraise the performance of the RDS sampling process against assumptions of the method, to generate a priori hypotheses about the potential implication of these patterns for interpreting the main trial findings as recommended by Gile et al. [4,5], to assess the extent to which the actual sampling process differed from the model. We will report our findings according to emerging STROBE guidelines for the reporting of RDS surveys. [6]

In this analysis, we focused on three main areas:
1) Indicators related to success of onward-recruitment and outcome data missingness.
2) Indicators related to random within-network recruitment, including evidence of seed dependency, and evidence of whether recruited participants appeared to be similar to reported characteristics of the underlying sex worker population (ego-nets).
3) Indicators related to network size (degree), that is the reported number of sex workers participants reported knowing, were eligible for the study and who they might have considered recruiting, which is used to calculate the RDS-II weights.

1. ONWARD-RECRUITMENT AND OUTCOME DATA MISSINGNESS

Method: First, we generated recruitment trees and calculated the proportion of recruiters who did not successfully recruit two recruit further participants, excluding the final wave. Second, we calculated the proportion of all sex workers recruited to the survey for whom primary outcome data (risk of HIV acquisition among HIV negatives and risk of HIV transmission among HIV positives) were unavailable. We examined these for each arm of the trial and compared between the AMETHIST intervention and standard of care arms.

Interpretation: All seeds were productive in onwards recruitment (Figure 3A). Very few recruited participants had missing data for the primary outcome, and there was no evidence of difference between arms in this at endline.

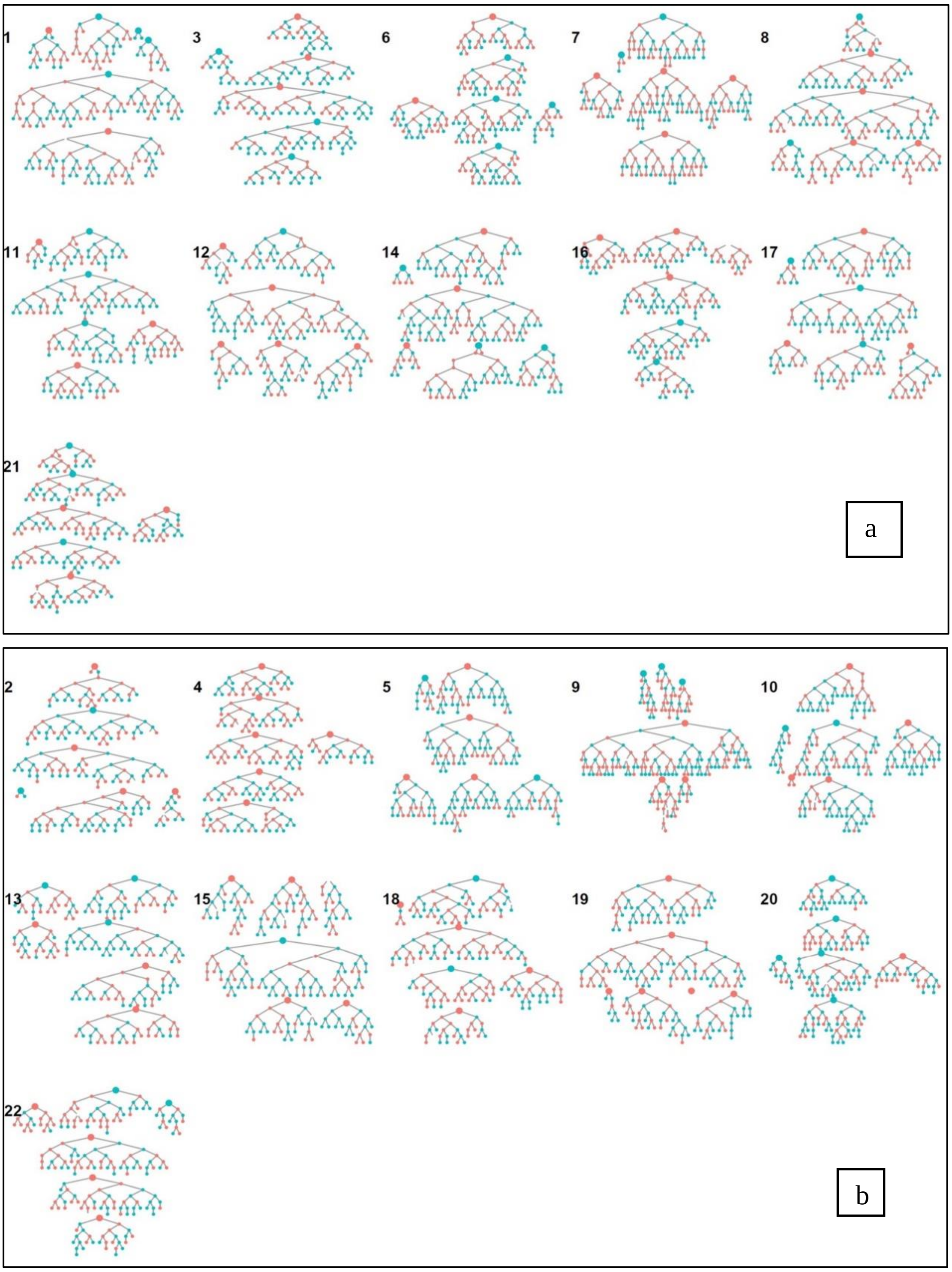

Figure A5: Recruitment tree diagrams in the usual care arm (a) and intervention arm (b). Participants are depicted by circles with their recruits shown as the connected circles below them. The larger circles denote seed participants. FSWs younger than 25 (red) and older than 25 (green).

2. RANDOM WITHIN-NETWORK RECRUITMENT

RDS studies have typically assessed the possible impact of biases on an estimated proportion through simulation of a social network and/or of the RDS process that runs over [X] it through there are a few examples where biases could be empirically tested [XI]. For our study, we are primarily interested in assessing whether deviation from the assumed recruitment process might be differential by intervention arm and thus differentially biasing site estimates and study findings. We therefore investigate assumptions as follows, by study arm.

Method: For each site and categorised by arm, we graphically examined the convergence of the primary outcome estimate over sample accumulation in order to assess whether the estimate appeared to have stabilized over successive waves of recruitment and whether it was independent of initial seed characteristics. The random walk model requires the assumption that the final estimates are no longer influenced by initial seed characteristics. If seed-specific estimates remain different from each other by the final sample wave, this observation might suggest that the population is split or extremely clustered into separate sub-groups rather than fully connected as the random walk model assumes [XII]. While graphical examination does not give an objective and definitive answer for whether an estimate has converged and is independent of seed characteristics, a simple test for convergence is not recommended [6]. For each site and each seed, categorised by arm, we graphed how the prevalence of the outcome changed with each recruited wave of the sample – Figure 4A – therefore combines both bottleneck and convergence plots as these are known in the RDS literature. We also asked sex workers to report on characteristics of the sex workers they knew and could have recruited to the study (their ego-net). We compared summary characteristics of the reported ego nets with characteristics of the recruited sample. Results are shown in Figure 4A (combined bottleneck and convergence plots for % of all FSW with the primary outcome, the risk of HIV acquisition/transmission) and Table 5A (RDS diagnostics).

Interpretation: Across all sites, it appeared that the estimate for the primary outcome (the risk of HIV acquisition/transmission) had achieved convergence (Figure 4A). The lack of divergence in seed specific estimates suggests that recruitment did not get caught within sub-networks related to the risk of HIV acquisition/transmission.

Most sex workers have good or better relationship with other female sex workers (74% in both arms). They tended to report that among sex workers they knew, a higher proportion were both under 25 and over 25 than the sample who were eventually recruited to the study (ratios of 0.63 and 0.90 for under 25; 0.80 and 0.74 for over 25, across usual care and treatment arm respectively). Sex workers also tended to report that less of the sex workers they knew were relatively new sex workers (<1 year in sex work) compared to the recruited sample, and that less had attended the clinic than the recruited sample. However, the proportions were similar across treatment arms. The proportion, among sex workers they knew, of sex workers who had heard of the Sisters programme is similar to that of the sample who were eventually

recruited to the study. A limitation of these comparisons is that it was not practically feasible to ask sex workers about each individual woman in their ego-net. While this gave us an indication of the composition of women's networks, it is not directly comparable with participant self-reports, which are measured as a continuous mean for each site. Sex workers familiar with the Sisters programme were similar amongst survey participants and reports of the proportion of sex workers in participants' ego-nets (ratios of 0.99 and 1.03 for ever hearing of the programme, across usual care and treatment arm respectively). This could suggest that RDS recruitment chains are not getting into a wider network of sex workers unfamiliar with the Sisters programme.

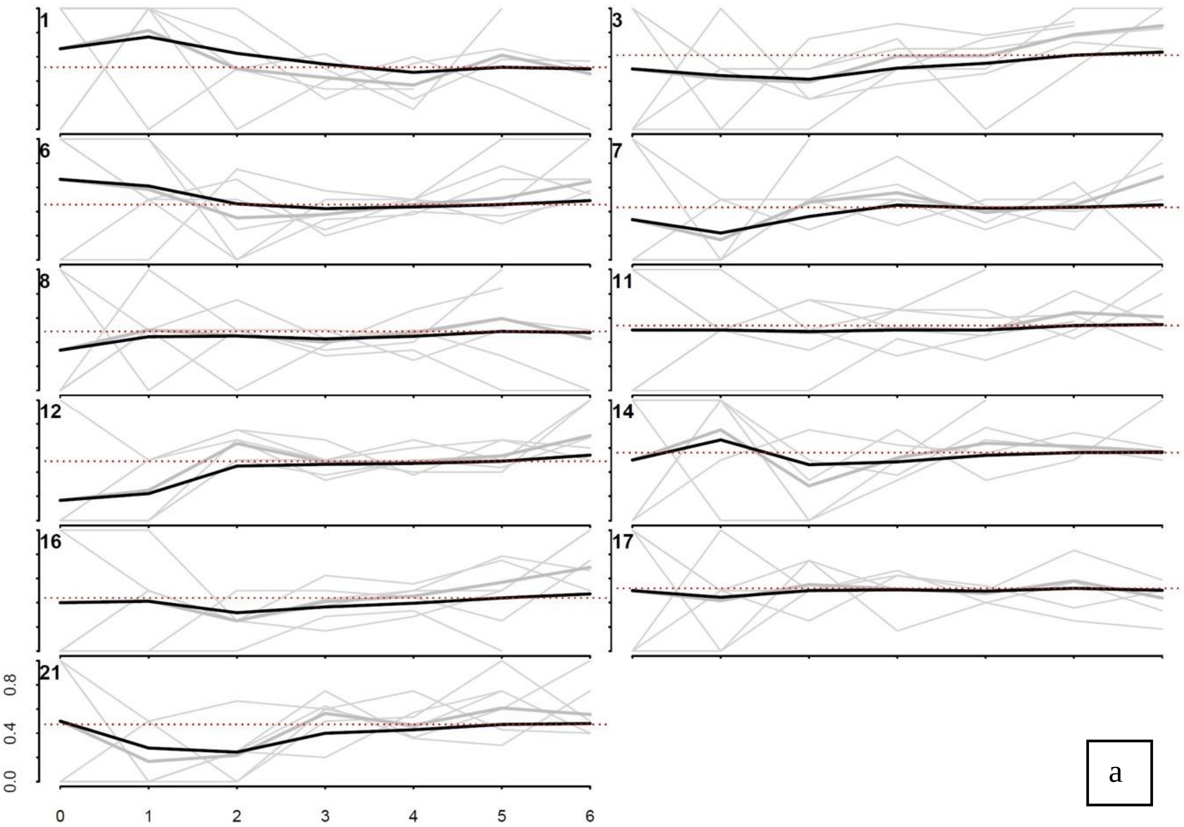

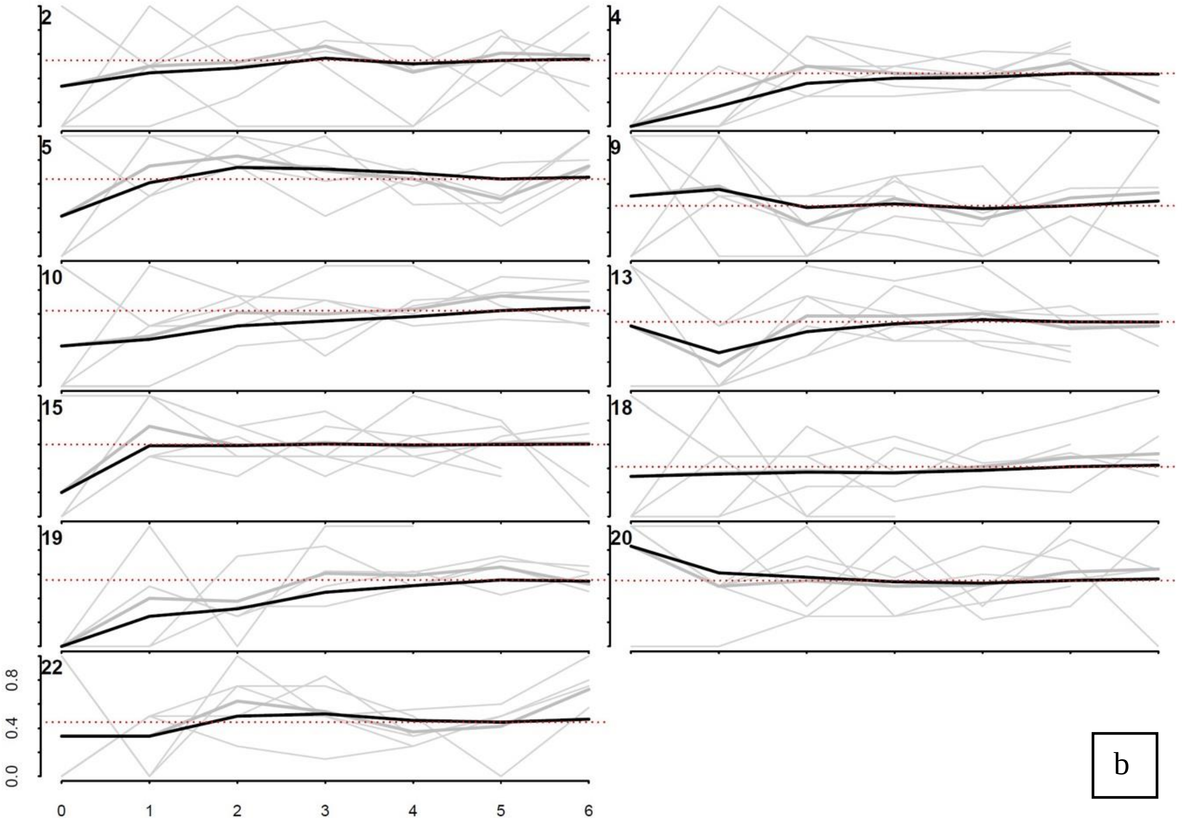

Figure A6: Convergence of the proportion of all female sex workers at risk of HIV acquisition (among HIV negatives) or transmission (among HIV positives) in 22 sites at endline; the usual care arm (a) and the intervention arm (b). The heavy black lines indicate the cumulative RDS-II weighted estimate overall for each site, while the grey lines are unweighted proportions for each seed, by sample wave.

Table A6: RDS diagnostic results

Characteristics	Mean of intervention site (min-max)	Mean of usual-care sites (min-max)	Mean difference
**Median network size**	3.00 (3.00, 4.50)	3.50 (2.00, 5.00)	-0.500
Proportion of good or very good relations	0.743 (0.672, 0.818)	0.741 (0.565, 0.893)	0.002
Ego-net proportion under 25 years old	0.180 (0.092, 0.305)	0.194 (0.069, 0.296)	-0.014
Sample proportion under 25 years old	0.286 (0.101, 0.515)	0.216 (0.091, 0.378)	0.070
Ratio of ego-net and sample proportion < 25	1.583 (1.099, 1.687)	1.111 (1.320, 1.278)	0.472
Ego-net proportion over 35 years old	0.576 (0.490, 0.719)	0.581 (0.417, 0.744)	-0.005
Sample proportion over 35 years old	0.714 (0.485, 0.899)	0.784 (0.622, 0.909)	-0.070
Ratio of ego-net and sample proportion > 35	1.241 (0.991, 1.250)	1.351 (1.490, 1.222)	-0.110
Ego-net proportion in SW > 1 year	0.621 (0.546, 0.774)	0.622 (0.544, 0.726)	-0.001
Sample proportion in SW > 1 year	0.907 (0.862, 0.943)	0.924 (0.852, 0.973)	-0.017
Ratio of ego-net and sample proportion in SW > 1 year	1.460 (1.579, 1.218)	1.485 (1.566, 1.340)	-0.025
Ego-net proportion heard of Sisters	0.845 (0.777, 0.909)	0.862 (0.766, 0.935)	-0.017
Sample proportion heard of Sisters	0.856 (0.559, 0.945)	0.841 (0.705, 0.909)	0.015
Ratio of ego-net and sample proportion heard of Sisters	1.013 (0.719, 1.039)	0.975 (0.920, 0.972)	0.037
Ego-net proportion attended clinic ever	0.830 (0.771, 0.920)	0.834 (0.737, 0.906)	-0.004
Sample proportion attended clinic in the last 12 months	0.570 (0.365, 0.715)	0.526 (0.368, 0.697)	0.044
Ratio of ego-net and sample proportion attended clinic	0.687 (0.474, 0.777)	0.631 (0.500, 0.769)	0.056

NETWORK SIZE

Method: RDS-2 estimates have been found to be sensitive to errors in reported degree, particularly in participants with low degrees whose status has a higher weight [VI]. RDS also assumes that all social contacts are reciprocated. RDS surveys have been used to characterise the underlying social network. The community mobilisation and adherence support activities might change the structure and composition of the FSW social networks in the intervention sites, possibly, and the RDS sampling process that runs over these networks might be differentially biased by the trial arm. In this RDS survey, however, it was non-differential by arm (Table A6).

Survey participants were asked to estimate the size of their network (and the extent to which recruitment might vary by relationship characteristics by asking participants about their relationship with their recruiter) in the interview. This reported network degree is used to weight outcomes in the RDS-2 analysis. This approach makes assumptions about the network sampling process in RDS: that participants randomly recruit from within their own network and any individual's chance of recruitment by a recruiter is inversely proportional to how many sex workers their recruiter knows; that the number of sex workers a woman reports she knows is equivalent to the number that know (and could have recruited) her 'reciprocity'; and that the overall size of the FSW population is large relative to the size of the recruited sample (because if the recruited population tends toward the complete number of potential recruits, all recruitment probabilities tend toward 1).

Bias could additionally be introduced through RDS-II's assumption of with-replacement sampling, when in fact sex workers are only ever sampled once. This bias can be greater when the sampling fraction is high. We assessed the potential effects of assuming with-replacement sampling by comparing out findings weighted using RDS-II with those weighted using RDS-SS for a range pf possible site populations sizes (250 and 500 per site) [6]. These findings are presented in Tables 7A1 – 7A3.

Interpretation: Network size reported in the interview was similar between treatment arms, ie., it was non-differential by arm and thus unlikely to bias the main study finding (3.00 [ranging between 3.00 and 4.50] versus 3.50 [ranging between 2.00 and 5.00] in the usual care and intervention arm, respectively). Compared to previous RDS [7]; the reported net-work degree is substantially lower. However, this is not differential by arm hence it's impact on the outcome seems minimal.

OVERALL INTERPRETATION OF RDS DIAGNOSTICS ANALYSES, AND RELEVANCE TO INTERPRETATION OF MAIN TRIAL FINDINGS.

Overall, we judged that the respondent driven sampling had worked well across the 22 sites. We made this judgement prior to conducting the primary outcome analysis, so as not to bias our interpretation.

Importantly, the estimate of the primary outcome appeared to have converged well implying good representativeness. There was little evidence that the RDS recruitment process might have differed by arm and therefore that differential bias resulting from the RDS weighting affected our study findings.

We did find differences in the characteristics of women described in participant's egonets and the women recruited to the study overall, particularly by age and by familiarity with the programme. However, there are challenges with measuring egonet characteristics efficiently. We asked participants to report rough proportions, which are not directly comparable to the individually self-reported participant characteristics.

Appendix **7: R CODE FOR PRIMARY OUTCOME**

```
# Main Functions
# New version of length which can handle NA's: if na.rm==T, don't count them
length2 <- function (x, na.rm=T) {
          if (na.rm) sum(!is.na(x))
            else       length(x)
}

# Add new rows for table appearance
insertRow <- function(existingDF, newrow, r) {
  existingDF[seq(r+1,nrow(existingDF)+1),] <- existingDF[seq(r,nrow(existingDF)),]
  existingDF[r,] <- newrow
  existingDF
}

# RDSII Weighted Summaries (by arm,cluster and categories of outcome)
# Inputs: data (data), outcome in character format (outcome)
rdsII.weightedsumms <-   function(data, outcome){
  results <- c()
  results <- data %>%
    # Apply the weighted mean function to each site individually, returning a vector of means
    plyr::ddply('sitenum', function(x) weighted.mean(x[,outcome], (x[,"network.size.variable"]), na.rm=TRUE)) %>%
    .[,2]
  return(results)
}

# Weighted cluster summaries for analysis using RDS 2; without cluster sizes analysis but only proportions:
# Inputs: data, and an outcome in character format (outcome).

clustersummendpts1 <- function(data, outcome){
  # Create a 22 row matrix for the cluster results
  design <- summaryBy(intervention ~ sitenum, data=data)
  # Merge the rds-weighted prevalences for each site, for endline
  results <- cbind(design, rdsII.weightedsumms(data, outcome))
  # Name the columns
  colnames(results) <- c("sitenum", "intervention", "obser.prob.weighted")
  return(results)
}
```

```
## Composite functions
#  Table row of cluster-summaries, num/denom., weighted-% for table
rdsII.weighted.summary.byarm    <- function(data, outcome){
  design    <- summaryBy(intervention ~ sitenum, data=data)
  # Cluster summaries for overall numerator and denominator
  ns        <- ddply(data, 'intervention', function(x) c(length2(x[,outcome]), sum(x[,outcome], na.rm=T)))
  # Genereate rdsified cluster summaries [Intervention]  #Catch missing data and remove
  data      <- subset(data, !is.na(data[,outcome]) & !is.na(data[,"network.size.variable"]))
  rdsified <- rdsII.weightedsumms(data, outcome)
  # Calculate mean, min, and max of cluster summaries by intervention status
  dp         <- "%.1f"
  rdsified1 <- subset(rdsified, design$intervention.mean==1)
  r          <- paste(sprintf("%.0f", ns[2,3]), "/",
                      sprintf("%.0f", ns[2,2]), "; ",
                      sprintf(dp, mean(rdsified1)*100),"% (",
                      sprintf(dp, min(rdsified1)*100), "%, ",
                      sprintf(dp, max(rdsified1)*100), "%)",
                      sep="")
  rdsified0 <- subset(rdsified, design$intervention.mean==0)
  s          <- paste(sprintf("%.0f", ns[1,3]), "/",
                      sprintf("%.0f", ns[1,2]), "; ",
                      sprintf(dp, mean(rdsified0)*100),"% (",
                      sprintf(dp, min(rdsified0)*100), "%, ",
                      sprintf(dp, max(rdsified0)*100), "%)",
                      sep="")

  # Calculate the mean difference
  p <- (mean(rdsified1)  - mean(rdsified0))*100
  q <- paste(sprintf("%.2f", p),"%", sep="")
  r <- cbind(r , s , q)
  return(r)
}
```

```
## Cluster analysis
# Adjusted analysis for primary and secondary outcomes
# Build Table 2
table.rdsII  <- matrix(NA, nrow = 0, ncol = 4)
newrow <- c('-','-','-','-')
for (var in c("outcome_composite",
              "outcome_hivneg",
              "outcome_hivpos"
)){
dd            <-   subset(d[, c('sitenum',      'wave',              'age',               'network.size.variable',
                               'intervention', 'outcome_hivpos',    'outcome_hivneg',    'outcome_composite'
                     )],)
  # Extract main variables to include in the analysis
  endline      <-  subset(dd, dd$wave!= 0)                 # And remove the seeds for the analysis itself
  results      <-  clustersummendpts1(endline, var)        # Create cluster summaries (using the ' clustersummendpts1' function, written above)
  # Adjusted analysis
  step1                        <-  glm(get(var) ~  age, family=binomial(link='logit'),  data=endline)  # Step 1: fit a model on cluster summaries to predict expected prevalence
  endline$adj.prob             <-  predict(step1, endline, type="response")                           # For each women, get predicted probability of outcome
  results$adj.prob.weighted    <-  rdsII.weightedsumms(endline, 'adj.prob')                            # Add the RDS II weighted probabilities to the results
  results$RD.residuals.weighted  <-  results$obser.prob.weighted - results$adj.prob.weighted           # Step 2: risk difference residuals, subtract observed prevalence from expected prevalence
  results$RR.residuals.weighted  <-  results$obser.prob.weighted / results$adj.prob.weighted           # Step 2: risk ratios residuals, divide observed number by expected number
  rdsII.adj.weighted           <-  lm(RD.residuals.weighted ~ intervention, data=results)              # Run regression to estimate risk differences

  # Create row for the tables
  # Raw numbers, weighted proportions, and adjusted and weighted risk difference
  assign(paste("table.rdsIIa"),
        cbind(paste(as.data.frame(rdsII.weighted.summary.byarm(endline, var))[,1]),
              paste(as.data.frame(rdsII.weighted.summary.byarm(endline, var))[,2]),
              paste(sprintf("%.1f", 100*rdsII.adj.weighted$coefficients["intervention"]),"% (",
                    sprintf("%.1f", 100*confint(rdsII.adj.weighted, "intervention")[1]),"%, ",
                    sprintf("%.1f", 100*confint(rdsII.adj.weighted, "intervention")[2]),"%)",
                    sep=""),
              sprintf("%.2f", summary(rdsII.adj.weighted)$coefficients[2,4])))
  table.rdsII <- rbind(table.rdsII, table.rdsIIa)
}
Table.rdsII.ad <- as.data.frame(table.rdsII)
row               <- matrix(, nrow=3, ncol=1)
Table.rdsII.ad    <- cbind(row, Table.rdsII.ad)
newrow            <- c('-','-','-','-','-')
Table.rdsII.ad    <- insertRow(Table.rdsII.ad, newrow, 1)
Table.rdsII.ad    <- insertRow(Table.rdsII.ad, newrow, 3)
Table.rdsII.ad[,1]  <- c("**Primary outcome**",
                        "Risk of transmission/acquisition",
                        "**Secondary outcomes**",
                        "Risk of aquisition among HIV -ves",
                        "Risk of transmission among HIV +ves")

colnames(Table.rdsII.ad)  <- c("", "Intervention n/N; mean RDS-adjusted proportions (min,max)",
                                   "Control n/N; mean RDS-adjusted proportions (min,max)",
                                   "age-Adjusted-Risk Difference (95% CIs)",
                                   "p-value")
```

APPENDIX 8: SENSITIVITY ANALYSES

Table A7.1: Effect estimates for the primary outcome using different sensitivity analyses

Primary outcome<br>Risk of transmission/acquisition	Intervention n/N; mean RDS-adjusted proportions (min,max)	Control n/N; mean RDS-adjusted proportions (min,max)	Adjusted-Risk Difference (95% CIs)	p-value
Adjusted for age and HIV prevalence at endline RDS II weighted	1156/2131; 55.3% (42.4%, 68.5%)	1104/2137; 52.7% (45.7%, 66.0%)	-2.7% (-4.8%, -0.5%)	0.02
Adjusted for age excluding Victoria Falls (a contaminated usual care site) RDS II weighted	1156/2131; 55.3% (42.4%, 68.5%)	1011/1941; 53.3% (45.7%, 66.0%)	-1.5% (-6.3%, 3.3%)	0.52
RDS II unweighted but adjusted for age (also including Victoria Falls)	1156/2131; 54.2% (42.9%, 66.7%)	1104/2137; 51.6% (45.9%, 64.3%)	-1.0% (-6.1%, 4.2%)	0.70
Among those attending Sisters clinic in the past year adjusted for age and RDS II weighted (also including Victoria Falls)	640/1298; 49.0% (37.7%, 64.8%)	523/1157; 46.0% (34.5%, 59.1%)	-0.3% (-6.1%, 5.4%)	0.90
RDS II weighted with successive sampling adjusted for age with different population size estimate*				
Population size = 250 per site	1197/2217; 55.0% (43.3%, 68.3%)	1151/2222; 52.8% (45.9%, 65.3%)	-2.1% (-7.4%, 3.2%)	0.42
Population size = 500 per site	1197/2217; 55.0% (43.3%, 68.3%)	1151/2222; 52.8% (45.9%, 65.3%)	-3.1% (-9.8%, 3.6%)	0.34
Adjusted for age and HIV prevalence at endline RDS II weighted with different cut-off for protective PrEP (TDF) level				
TDF =350 fmol/punch	1133/2131; 54.3% (42.4%, 67.7%)	1093/2137; 52.4% (45.0%, 65.3%)	-1.6% (-6.4%, 3.3%)	0.51
TDF = any detectable level	1081/2131; 51.9% (38.1%, 65.2%)	1059/2137; 50.5% (41.8%, 63.9%)	-2.1% (-7.3%, 3.1%)	0.41

*No data was removed, ie, all data including seeds were used to construct the RDS

Table A7.2: Effect estimates for the secondary outcome among HIV positives using different sensitivity analyses

**Secondary outcome** Risk of transmission	Intervention n/N; mean RDS-adjusted proportions (min, max)	Control n/N; mean RDS-adjusted proportions (min, max)	Adjusted-Risk Difference (95% CIs)	p-value
Adjusted for age and HIV prevalence at endline RDS II weighted	63/931; 5.8% (1.9%, 16.0%)	103/1041; 10.4% (4.8%, 15.4%)	-5.5% (-8.2%, -2.9%)	0.00
Adjusted for age excluding Victoria Falls (a contaminated usual care site) RDS II weighted	63/931; 5.8% (1.9%, 16.0%)	94/943; 10.4% (4.8%, 15.4%)	-5.6% (-8.4%, -2.8%)	0.00
RDS II unweighted but adjusted for age (also including Victoria Falls)	63/931; 6.9% (3.4%, 14.8%)	103/1041; 9.8% (4.1%, 16.4%)	-3.9% (-6.8%, -1.1%)	0.01
Among those attending Sisters clinic in the past year adjusted for age and RDS II weighted (also including Victoria Falls)	40/638; 5.6% (0.5%, 16.9%)	55/643; 8.6% (2.0%, 15.2%)	-3.8% (-7.6%, 0.1%)	0.05
RDS II weighted with successive sampling adjusted for age with different population size estimate*				
Population size = 250 per site	63/971; 5.6% (1.8%, 15.8%)	106/1077; 10.4% (4.8%, 15.2%)	-3.8% (-7.0%, -0.7%)	0.02
Population size = 500 per site	63/971; 5.6% (1.8%, 15.8%)	106/1077; 10.4% (4.8%, 15.2%)	-3.2% (-7.3%, 0.9%)	0.12

*No data was removed, ie, all data including seeds were used to construct the RDS

Table A7.3: Effect estimates for the secondary outcome among HIV negatives using different sensitivity analyses

**Secondary outcome** Risk of acquisition	Intervention n/N; mean RDS-adjusted proportions (min, max)	Control n/N; mean RDS-adjusted proportions (min, max)	Adjusted-Risk Difference (95% CIs)	p-value
Adjusted for age and HIV prevalence at endline and RDS II weighted	1093/1200; 92.1% (83.6%, 97.3%)	1001/1096; 92.2% (84.9%, 96.6%)	-0.6% (-4.6%, 3.4%)	0.74
Adjusted for age excluding Victoria Falls (a contaminated usual care site) and RDS II weighted	1093/1200; 92.1% (83.6%, 97.3%)	917/998; 92.9% (86.7%, 96.6%)	-1.3% (-5.2%, 2.5%)	0.48
RDS II unweighted but adjusted for age (also including Victoria Falls)	1093/1200; 91.2% (83.1%, 96.6%)	1001/1096; 91.2% (85.3%, 95.4%)	-0.6% (-4.4%, 3.3%)	0.77
Among those attending Sisters clinic in the past year adjusted for age and RDS II weighted (also including Victoria Falls)	600/660; 90.9% (78.3%, 98.8%)	468/514; 90.9% (80.3%, 98.3%)	-0.1% (-5.5%, 5.2%)	0.96
RDS II with successive sampling adjusted for age, with different population size estimate*				
Population size = 250 per site	1134/1246; 92.2% (84.1%, 97.4%)	1045/1145; 92.2% (86.3%, 96.5%)	-1.5% (-5.3%, 2.4%)	0.43
Population size = 500 per site	1134/1246; 92.2% (84.1%, 97.4%)	1045/1145; 92.2% (86.3%, 96.5%)	-2.7% (-7.6%, 2.3%)	0.27
Adjusted for age and HIV prevalence at endline RDS II weighted with different cut-off for protective PrEP level				
TDF =350 fmol/punch	1070/1200; 90.4% (82.4%, 95.9%)	990/1096; 91.5% (83.2%, 96.4%)	-1.9% (-5.9%, 2.0%)	0.33
TDF = any detectable level	1018/1200; 86.0% (77.1%, 95.0%)	956/1096; 87.9% (80.6%, 94.6%)	-2.8% (-7.1%, 1.5%)	0.19

*No data was removed, ie, all data including seeds were used to construct the RDS

REFERENCES

1. Cowan FM, Machingura F, Chabata ST, Ali MS, Busza J, Steen R, et al. Differentiated prevention and care to reduce the risk of HIV acquisition and transmission among female sex workers in Zimbabwe: study protocol for the 'AMETHIST' cluster randomised trial. *Trials*. 2022;**23**(1):209. doi: 10.1186/s13063-022-06119-w.
2. Volz, E. and D.D. Heckathorn, Probability Based Estimation Theory for Respondent Driven Sampling. *Journal of Official Statistics*, 2008. **24**(1): p. 79-97.
3. Hayes, J.R. and L.H. Moulton, Cluster Randomised Trials. 2009: Chapman and Hall/CRC.
4. Gile KJ, Johnson LG, Salganik MJ. Diagnostics for respondent driven sampling. *Journal of the Royal Statistical Society: Series A*, 2015; **178**(1): p. 241-269.
5. Gile KJ. Improved inference for respondent-driven sampling data with application to HIV prevalence estimation. *Journal of the American Statistical Association* 2011; **106**(493): p. 135-146.
6. White, R.G., et al., Strengthening the Reporting of Observational Studies in Epidemiology for respondent-driven sampling studies: "STROBE-RDS" statement. Journal of clinical epidemiology, 2015. **68**(12): p. 1463-1471.
