# Supplementary material for: A risk-differentiated, community-led intervention to strengthen uptake and engagement with HIV prevention and care cascades among female sex workers in Zimbabwe (AMETHIST): a cluster randomised trial
Source: Lancet Glob Health. 2024 Aug 14;12(9):e1424–35. doi: 10.1016/S2214-109X(24)00235-3 (PMC11345450; doi:10.1016/S2214-109X(24)00235-3)
Supplement: Equitable Partnership Declaration [file mmc4.pdf]

THE LANCET
Global Health

## Supplementary appendix 4

This Equitable Partnership Declaration (EPD) was submitted by the authors, and we reproduce it as supplied. It has not been peer reviewed. *The Lancet*'s editorial processes have not been applied to the EPD.

Supplement to: Cowan FM, Machingura F, Ali MS, et al. A risk-differentiated, community-led intervention to strengthen uptake and engagement with HIV prevention and care cascades among female sex workers in Zimbabwe (AMETHIST): a cluster randomised trial. *Lancet Glob Health* 2024; **12:** e1424–35.

**Appendix 4**

**AMETHIST Trial Equitable Partnership Declaration**

| |
|---|
| **a) Study design:**<br>The study was co-designed by Zimbabwean and UK based researchers (led by Dr Fortunate Machingura in Zimbabwe), Zimbabwean programme staff (led by Primrose Matambanadzo), Zimbabwean sex workers, Zimbabwean Ministry of Health and National AIDS Council staff.<br><br>Frances Cowan is overall PI of the collaborative award, is based full time in Zimbabwe and was Director of CeSHHAR Zimbabwe throughout the trial. |
| **b) Clinical study processes:**<br>The Key Programmes research lead was Dr Fortunate Machingura – who led design and day to day implementation of all research activities. She is the Zimbabwean PI and qualitative research lead (supported by Joanna Busza LSHTM UK).<br>Ms Primrose Matambanadzo is the programme lead and over saw all aspects of intervention delivery and reporting. |
| **c) Data interpretation:**<br>Quantitative data: Sungai Chabata (CeSHHAR Zimbabwe) is the lead Zimbabwe statistician for the trial, worked with Prof James Hargreave (LSHTM) and Prof Andrew Phillips (UCL) on sample size calculations, implementation of randomisation procedures and drawing up the statistical analysis plan. He undertook all RDS diagnostics and supported Dr Sanni Ali (LSHTM) to undertake the primary outcomes analysis.<br>Programme data: Mr Jeffrey Dirawo is CeSHHAR IT lead and oversaw collection and curation of all programme data. Albert Takura undertook continuous analysis of programme data to support programmatic<br>Qualitative data – Dr Machingura leads a team of Zimbabwean social scientists who collected, transcribed and translated and are analysing all qualitative data (supported by Joanna Busza LSSHTM) |
| **d) Manuscript preparation:**<br>The analysis plan was drafted by Sungai Chabata and all co-investigators commented.<br>The manuscript was drafted by Frances Cowan, Fortunate Machingura, Sungai Chabata, Sanni Ali, Primrose Matambanadzo, James Hargreaves and Andrew Phillips. |

1. Were the data used in your study collected by authors named on the paper, or have they been extracted from a source such as a national survey? ie, is this a secondary analysis of data that were not collected by the authors of this paper. If the authors of this paper were not involved in data collection, how were data interpreted with sufficient contextual knowledge?

   The Lancet Global Health *believe contextual understanding is crucial for informed data analysis and interpretation.*

| |
|---|
| The data used in the study were collected by the authors named on the paper and the large research and implementation teams that they manage. |

2. How was funding used to remunerate and enhance the skills of researchers and institutions based in the area(s) of study? And how was funding used to improve research infrastructure in the area of study?

   *Potentially effective investments into long-term skills and opportunities within institutions could include training or mentorship in analytical techniques and manuscript writing, opportunities to lead all or specific aspects of the study, financial remuneration rather than requiring volunteers, and other professional development and educational opportunities.*

   *Improvements to research infrastructure could be funding of extended trial designs (such as platform trials) and use of master protocols to enable these designs, establishment of long-term contracts for research staff, building research facilities, and local control of funding allocation.*

| |
|---|
| **Skills:**<br>Annual AMETHIST consortium meetings where data were presented and interpretation was discussed in depth. Monthly data working group meetings (virtual) where quantitative analyses were planned, designed, presented and discussed. Regular data working sub-group meetings with where analysis and planning of individual papers was moved forward. Close working relationship with the members of the Measurement and Surveillance of HIV epidemics (MeSH) consortium including attendance at MeSH symposium and virtual symposia at international conferences. Qualitative working groups (held in person every 6 months with more frequent virtual meetings ) data presentation interpretation and paper writing. Conference attendance with opportunities to submit abstracts and present data as posters or oral presentations (CROI, IAS, IUSTI, ICASA, Zimbabwe National AIDS conference). All community cadres working on the project were reimbursed for their time in line with national guidance. Training of programme staff (including sex worker microplanners) to implement microplanning and train others to do so (the team has since trained teams in Zambia and Mozambique on behalf of MSF and UNAIDS). Monthly virtual meeting of programme implementation and data staff with Richard Steen (consultant guiding delivery of microplanning). |
| **Research infrastructure:**<br>Two thirds of funding for the Wellcome Trust Collaborative Award was spent in southern Africa. In Zimbabwe the award was used to strengthen the KP programme implementation research platform which has facilitated numerous nested research studies since 2011. The grant paid for video conferencing facilities at CeSHHAR to facilitate adequate communication both within Zimbabwe and internationally during the CV19 pandemic. |

3. How did you safeguard the researchers who implemented the study?

   *Please describe how you guaranteed safe working conditions for study staff, including provision of appropriate personal protective equipment, protection from violence, and prevention of overworking.*

| |
|---|
| CeSHHAR has comprehensive policies defining work practices across the organisation which relate to safeguarding (both of staff and research participants), dignity at work, travel policies which prohibit driving after dark (road traffic accidents as a serious risk in Zimbabwe). Implementation of these policies is closely monitored. The organisation is well known in communities and has |

worked across Zimbabwe since 2009. CeSHHAR has MoUs with all district councils in which it works and meets regularly with key stakeholders including political and traditional leadership and police. We conducted intensive community sensitisation prior to the surveys taking place.

*Benefits to the communities and regions of study*

4. How does the study address the research and policy priorities of its location?

*How were the local priorities determined and then used to inform the research question? Who decided which priorities to take forward? Which elements of the study address those priorities?*

Zimbabwe has one of the most severe HIV epidemics globally. HIV is a major priority for Zimbabwe's Ministry of Health and Child Care. Female sex workers remain disproportionately burdened by HIV. The need to engage female sex workers in HIV prevention and care is specifically highlighted in Zimbabwe's various National AIDS Strategic Plans (issued every 5 years since 2006). The Director of AIDS and TB Unit in the MoHCC and Head of Monitoring and Evaluation at NAC were both co-investigators of the trial. Importantly CeSHHAR has been at the forefront of establishing Zimbabwe's National Key Populations forum where this research has been presented at early stages of planning and development and results since disseminated. All Provincial and district medical officers and National AIDS Council officers working in study districts to part in the public randomisation meeting.

5. How will research products be shared in the community of study?

*For instance, will you be providing written or oral layperson summaries for non-academic information sharing? Will study data be made available to institutions in the region(s) of study?* The Lancet Global Health *encourages authors to translate the summary (abstract) into relevant languages after paper editing; do you intend to translate your summary?*

We have disseminated results at Key Population forum meetings (ie to community members, key population programme implementers and CBOs), local scientific meetings held by National AIDS Council, Zimbabwe Medical Association and University of Zimbabwe Annual Research Day. We will translate the summary into Shona and Ndebele and make this widely available (although many meetings are conducted in English).

6. How were individuals, communities, and environments protected from harm?

a) *How did you ensure that sensitive patient data was handled safely and respectfully? Was there any potential for stigma or discrimination against participants arising from any of the procedures or outcomes of the study?*

CeSHHAR has stringent data collection, handling and storage processes in place. Data are collected electronically in the field and immediately encrypted on saving before uploading to a secure cloud facility. The survey participants were female sex workers, they were surveyed in the

community at a venue selected by FSW representatives as safe and anonymous. There is the potential for deductive disclosure although no instances of this have been reported. All research staff are trained in GCP and in working with key populations. The procedures included finger prick blood sampling, venepuncture and taking of self collected vaginal swabs, all of which were acceptable to women. Women could opt out of procedures if they chose to do so. Very few <2% opted to do so.

b) *Might any of the tests be experienced as invasive or culturally insensitive?*

*The tests were blood tests and self conducted vaginal swabs. These are commonly performed and while minimally invasive are not considered culturally insensitive.*

c) *How did you determine that work was sensitive to traditions, restrictions, and considerations of all cultural and religious groups in the study population?*

*This survey was with sex workers. CeSHHAR has a long history of both providing services and conducting research in this population and is widely trusted in the sex worker community. Sex workers were actively involved in questionnaire development and piloting to ensure understandability and acceptability.*

d) *Were biowaste and radioactive waste disposed of in accordance with local laws?*

*Yes*

e) *Were any structures built that would have impacted members of the community or the environment (such as handwashing facilities in a public space)? If so, how did you ensure that you had appropriate community buy-in?*

*No*

f) *How might the study have impacted existing health-care resources (such as staff workloads, use of equipment that is typically employed elsewhere, or reallocation of public funds)?*

*The study provided additional funding for the intervention in trial communities. Microplanning has since been scaled up nationally through programmatic funding from GFATM and USAID.*

7. Finally, please provide the title (eg, Dr/Prof, Mr/Mrs/Ms/Mx), name, and email address of an author who can be contacted about this statement. This can be the corresponding author.

**Name:** Prof Frances M Cowan
**Email:** frances.cowan@lstmed.ac.uk
